# Untethered Micro/Nanorobots for Remote Sensing: Toward Intelligent Platform

**DOI:** 10.1007/s40820-023-01261-9

**Published:** 2023-11-30

**Authors:** Qianqian Wang, Shihao Yang, Li Zhang

**Affiliations:** 1 Jiangsu Key Laboratory for Design and Manufacture of Micro-Nano Biomedical Instruments, School of Mechanical Engineering, Southeast University, Nanjing, 211189 People’s Republic of China; 2 Department of Mechanical and Automation Engineering, The Chinese University of Hong Kong, Shatin, Hong Kong, 999077 People’s Republic of China; 3 Chow Yuk Ho Technology Centre for Innovative Medicine, The Chinese University of Hong Kong, Shatin, Hong Kong, 999077 People’s Republic of China; 4 T Stone Robotics Institute, The Chinese University of Hong Kong, Shatin, Hong Kong, 999077 People’s Republic of China; 5 Department of Surgery, The Chinese University of Hong Kong, Shatin, Hong Kong, 999077 People’s Republic of China

**Keywords:** Micro/nanorobot, Remote sensing, Wireless control, Self-propulsion, Actuation at small scales

## Abstract

Untethered micro/nanorobots that can wirelessly control their motion and deformation state have gained enormous interest in remote sensing applications due to their unique motion characteristics in various media and diverse functionalities. Researchers are developing micro/nanorobots as innovative tools to improve sensing performance and miniaturize sensing systems, enabling in situ detection of substances that traditional sensing methods struggle to achieve. Over the past decade of development, significant research progress has been made in designing sensing strategies based on micro/nanorobots, employing various coordinated control and sensing approaches. This review summarizes the latest developments on micro/nanorobots for remote sensing applications by utilizing the self-generated signals of the robots, robot behavior, microrobotic manipulation, and robot-environment interactions. Providing recent studies and relevant applications in remote sensing, we also discuss the challenges and future perspectives facing micro/nanorobots-based intelligent sensing platforms to achieve sensing in complex environments, translating lab research achievements into widespread real applications.

## Highlights

- A systematic review of latest developments of untethered micro/nanorobots-based remote sensing systems with an emphasis on designing new coordinated control and sensing approaches.
- The propulsion/motion control, functionalization of micro/nanorobots, sensing mechanisms, and applications are reviewed based on the up-to-date works.
- The design and application of micro/nanorobot-based sensing platforms are discussed with the goal of building intelligent remote sensing systems.

## Graphical abstract

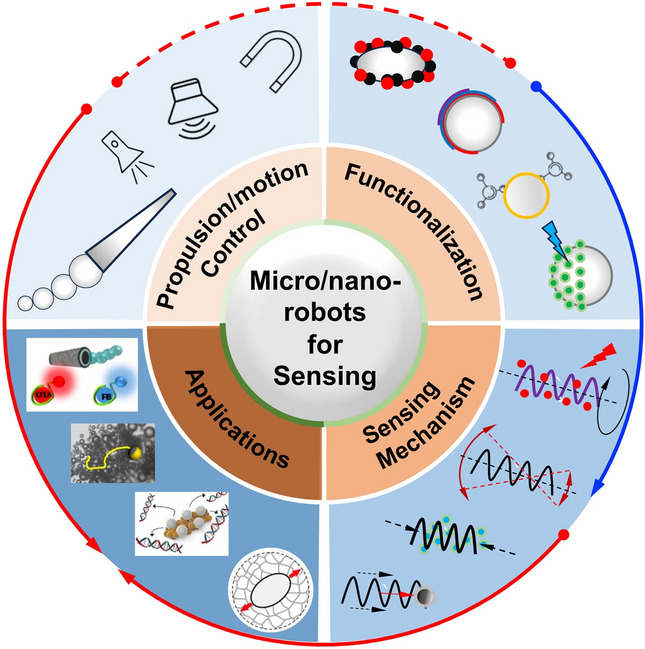

## Introduction

Hazardous pollutants are released into the environment due to the increasing anthropogenic activity and rapid industrialization. Exposure to these substances can be highly toxic, potentially fatal to humans, and harm the aquatic ecosystem. Thus, methods to sense and detect these pollutants, such as bacterial endotoxin, virus, and heavy metal ions, become essential in food safety and health monitoring. Detection techniques provide information on the presence and concentration of the target analyte (higher than the limit of detection), while sensing methods monitor changes in the target analyte under varying conditions. Conventional methods typically involve direct evaluation of specific toxins (*e.g.*, mass spectrum) or indirect detection using probe techniques (*e.g.*, DNA probes). These methods usually require sampling, extensive specimen purification, and qualified personnel to operate advanced instruments [1, 2]. In the case of hard-to-reach and easily contaminated samples, in situ sensing is advantageous, particularly for real-time monitoring of rapidly changing properties of the target analytes. Besides, active operation-based sensing, such as local mechanical properties measurement of cells and tissues, finds hard to conduct when applying conventional sensing strategies. In recent years, the rapid development of control systems and functionalization technology on micro/nanoscale objects has greatly promoted the application of micro/nanomachines in sensing tasks [3–8]. Consequently, this advancement leads to improved sensing resolution and accuracy as well as the miniaturization of sensing systems, enabling effective substances detection that traditional sensing methods find hard to achieve [9–13].

Untethered micro/nanorobots have come to the forefront as advanced tools due to their wireless operation capabilities and diverse functionalities. They are defined as mobile miniature devices in dimensions from several micro-/nanometer to millimeter scale that are capable of performing tasks in a controlled manner. Micro/nanorobots employ a variety of propulsion mechanisms, broadly classified into two main types: self-propulsion and external field-propulsion [14, 15]. Self-propelled microrobots derive their power from interactions with the surrounding environment, typically by catalyzing the breakdown of chemical fuels or through their self-degradation [16]. Their propulsion is specifically achieved through various strategies, including bubble generation [17], self-diffusiophoresis [18], self-electrophoresis [19, 20], Marangoni effect [21], and self-thermophoresis [22]. Micro/nanorobots can also be powered by external fields, such as magnetic field [23, 24], acoustic field [25, 26], electric field [27, 28], and light [29, 30]. Field-driven micro/nanorobots are designed with materials or structures that respond to external fields, thereby eliminating the need for chemical fuels in their environment [31, 32]. Combining different propulsion methods to develop hybrid-driven micro/nanorobots represents a promising approach for overcoming the limitations of a single propulsion method and expanding application scenarios [33–36]. Due to the synergistic effect of their motion characteristics and inherent physicochemical properties, micro/nanorobots find wide application in micromanipulation and targeted delivery [37–42].

Recently, micro/nanorobots have garnered significant attention as promising candidates for remote sensing due to their autonomous motion capabilities in various media, including solutions, biofluids, and even intracellular matrices [43–45]. Their small size makes them especially suitable as mobile sensors in complex media, and their controlled motion ability also contributes to micromixing, which is primarily governed by passive diffusion due to the laminar nature of flows at low Reynolds numbers [46]. The mobility of micro/nanorobots has demonstrated their capability in facilitating efficient micromixing and improving mass transfer, surpassing the limitations imposed by passive diffusion [47, 48]. However, these tiny robots are often restricted to specific operations or limited in certain application scenarios. It is essential to further functionalize them to meet a wide range of control and sensing requirements [49]. Combined with their motile features, functionalized micro/nanorobots can achieve enhanced performance due to real-time sensing capabilities and accelerated ‘on-the-fly’ reactions. Besides, micro/nanorobots-based sensing systems or platforms can perform in situ sensing in various media and even in cells [50, 51], thereby avoids sampling processes that might introduce errors in sensing precision and accuracy. Taking advantage of the mobile sensing capability, real-time bio-sensing and in vivo detection can be implemented in a controlled manner.

## Self-generated Signal-based Sensing

**Fig. 1:**
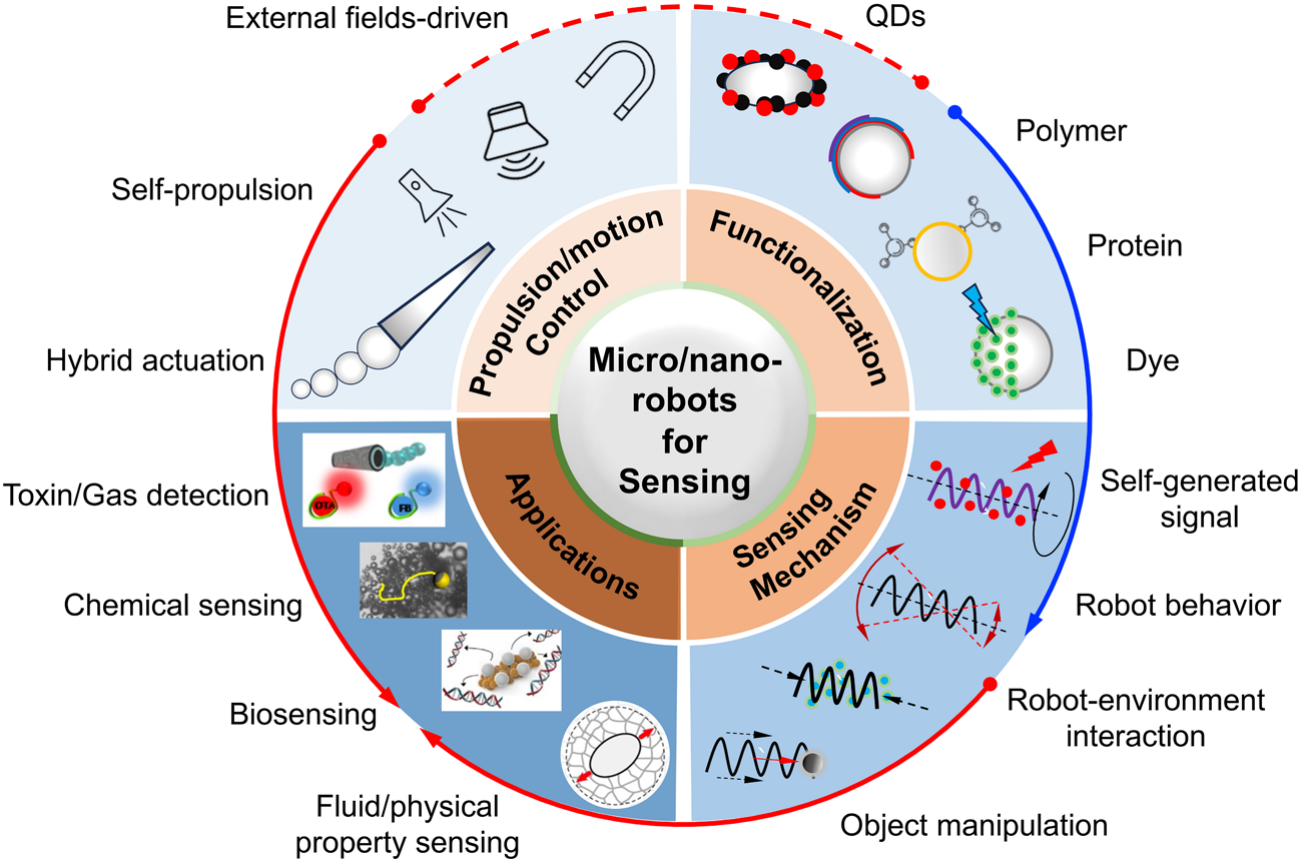
The outline of this review from four critical and closely related factors, including the propulsion/motion control of micro/nanorobots, functionalization of robots, sensing mechanism, and applications. Figures are adapted from the following references: toxin/gas detection [52], copyright (2017) American Chemical Society; chemical sensing [53], copyright (2019) American Chemical Society; biosensing [54], copyright (2022) Elsevier; fluid/physical property sensing [55], copyright (2016) Springer Nature

**Fig. 2:**
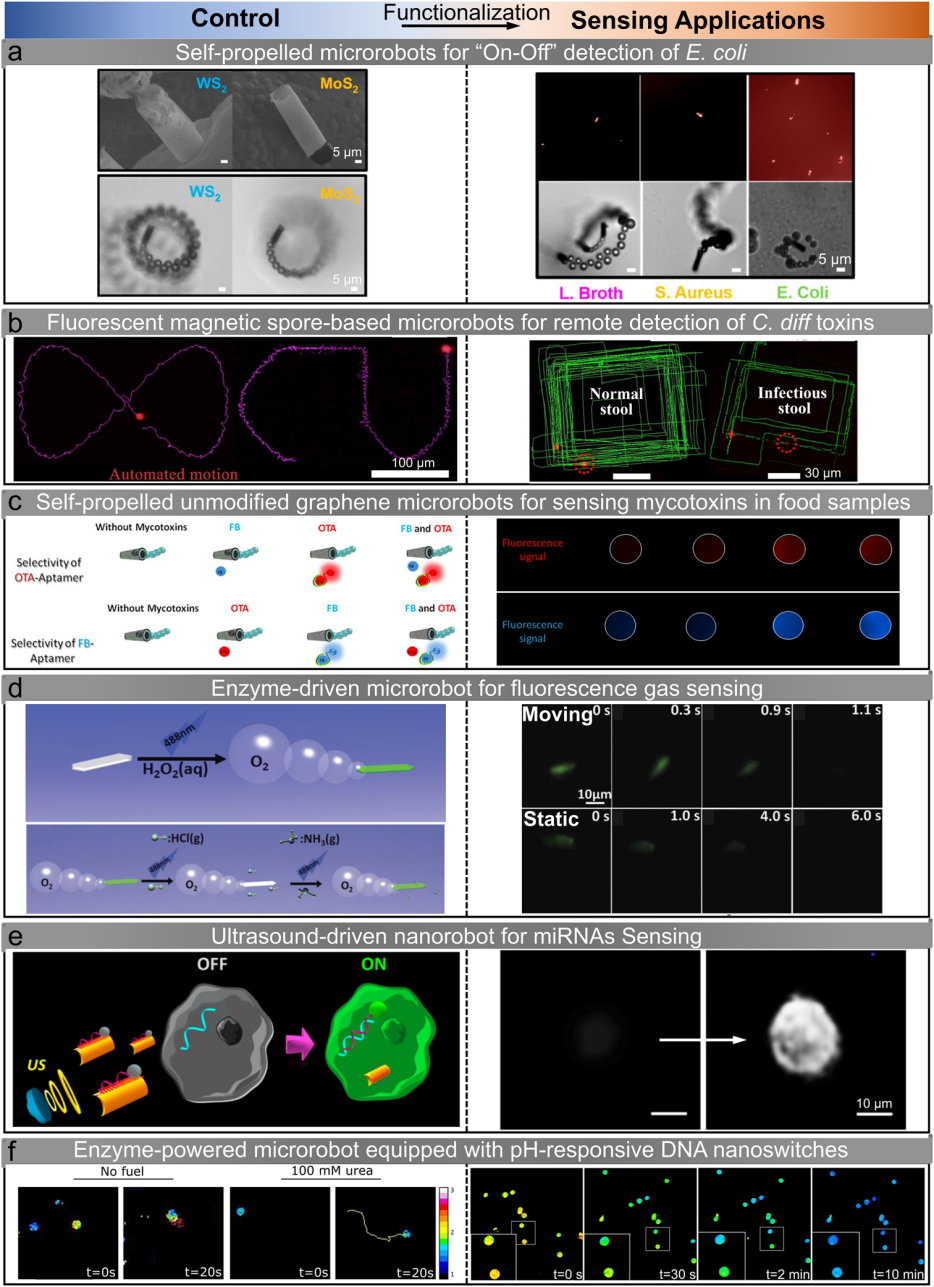
Self-generated signal-based sensing. **a** Peptide-modified self-propelled WS_2_/Pt and MoS_2_/Pt microrobots for on–off detection of *E. coli* [56]. Copyright (2020) American Chemical Society. **b** Fluorescent magnetic spore-based microrobots for remote detection of *C. diff* toxins [57]. Copyright (2020) The Authors. **c** Self-propelled unmodified graphene microrobots for quantitative analysis of mycotoxins in food samples [52]. Copyright (2017) American Chemical Society. **d** Enzyme-driven microrobot for fluorescence HCl and NH_3_ gases sensing [58]. Copyright (2016) Royal Society of Chemistry. **e** Ultrasound-driven nanorobot for single cell real-time miRNAs sensing [59]. Copyright (2015) American Chemical Society. **f** Self-sensing enzyme-powered microrobot equipped with pH-responsive DNA nanoswitches [60]. Copyright (2019) American Chemical Society

Self-generated signal-based sensing relies on detecting signals generated by the robot, such as on–off fluorescence detection of toxins, on–off luminescence for chemical sensing, and electrochemiluminescent for biosensing (Fig. 2). This type of sensing or detection mechanism typically depends on the functionalization of robots, with a particular emphasis on surface functionalization, given that chemistry plays a pivotal role in determining the physicochemical properties of micro/nanorobots. The surfaces can be modified to function as reaction sites, thereby significantly influencing the interaction between micro/nanorobots and various analyte molecules in the environment (Fig. 1).

### Toxin Sensing

In toxin detection, micro/nanorobots are commonly used for the detection of substances such as ricin B, Escherichia coli (*E. coli*), Clostridium difficile (*C. diff*), and others. Aptamer-mediated catalytic microrobots have been proposed for real-time on–off fluorescent detection of ricin B toxin [61]. The motion capability is achieved by designing the robot body using reduced graphene oxide (rGO)/Pt and synthesized through a standard membrane-template electrodeposition protocol. The Pt component of the microrobot serves as a catalyst for the decomposition of hydrogen peroxide (H_2_O_2_) in the solution, generating oxygen bubbles that create a recoil force propelling the microrobot. These microrobots are modified with a specific ricin B aptamer tagged with a fluorescein-amidine (FAM) dye, and the fluorescence emitted by the FAM dye is effectively quenched due to the π–π interactions with the rGO surface. In contrast to conventional binding processes, a moving microrobot accelerates the specific binding of the toxin to the dye conjugate, resulting in real-time fluorescent “On" detection, demonstrating the efficiency of microrobot-based sensing. “On-the-fly" sensing platforms are also proposed for detecting *E. coli* in bioassays. WS_2_/Pt and MoS_2_/Pt microrobots exhibit bubble-propulsion in 2% H_2_O_2_ solution, and they are functionalized with affinity peptide probe for on–off toxin detection [56]. The sensing performance is significantly influenced by the distinct surface characteristics of the microrobots. Due to improved peptide probe loading and release, WS_2_/Pt microrobot with a comparatively higher outer surface exhibits a 3.5-fold increase in sensitivity compared to MoS_2_/Pt microrobot (Fig. 2a). Therefore, the peptide-modified WS_2_/Pt microrobots are employed as a cost-effective sensing tool for the high-throughput determination of *E. coli* endotoxin, with a detection limitation of 1.9 ng mL^−1^. In addition to the tubular-shaped microrobot-based sensing method, a spherical fluorophore fluoresceinamine (FLA)/silica-NH_2_/Pt microrobot is proposed for “on-the-fly" sensing of sarin and soman simulants, based on the on–off fluorescent strategy [62]. The motion of multiple microrobots within a contaminated sample induces continuous mixing, significantly enhancing mass transfer. As a result, the reaction rates between the contaminated solution and the microrobots are increased compared to static microrobot counterparts. Moreover, the mobility of FLA-coated microrobots facilitates their real-time fluorescence quenching upon interaction with reactive nerve agents, enabling rapid and dynamic response.

In addition to the self-propelled microrobot-based sensing strategy, fluorescent magnetic spore-based microrobots have been designed as mobile sensors for detecting toxins secreted by *C. diff* in patients’ stool [57]. The synthesis procedure involves the direct deposition of magnetic nanoparticles and the encapsulation of sensing probes on porous natural spores, specifically magnetic Fe_3_O_4_ nanoparticles for actuation and functionalized carbon nanodots for selective fluorescence sensing (Fig. 2b). The fabricated paramagnetic spore-based microrobot becomes magnetized along its long axis when an external magnetic field is applied and demonstrates three motion modes: spinning, rotation-translation, and tumbling, depending on different magnetic field parameters. The microrobot can autonomously navigate along a pre-determined path by controlling the input magnetic field, and its fluorescence trajectories are recorded. Thus, real-time evaluation of *C. diff* toxins is accomplished by directly monitoring the fluorescence change. Compared with static sensing methods, the continuous movement in microrobots leads to an enhanced detection ability by facilitating a significant increase in the mass transfer of detected toxins. Such a quantum dots (QDs)-based microrobotic sensing strategy is also proposed using a magnetocatalytic hybrid Janus microrobot [63]. One side of the microrobot is modified with a large number of magnetic Fe_3_O_4_ and Pt nanoparticles, which endows the microrobot with magnetism and catalytic properties, respectively. This hybrid actuation design (two active areas) allows the microrobot to perform effective propulsion in the presence of H_2_O_2_ solution or magnetic fields. The sensing mechanism for lipopolysaccharides (LPS) relies on graphene QDs-based fluorescence quenching: the quenching effect occurs due to the highly specific recognition ability of the phenylboronic acid tags, which serve as receptors for the core polysaccharide region of the LPS. This strategy has been validated for the screening of complex urine and human serum samples.

Taking advantage of the mobile and high-sensitivity features, self-propelled microrobots have been utilized for food-safety diagnosis. The continuous movement of rGO/nickel/platinum nanoparticle microrobots around the sample enhances mixing and increases the contacts between targets and receptors [64]. The outer rGO layer and the internal Pt nanoparticle layer equip the microrobot with adsorption and catalytic propulsion features, enabling the on-the-move capture of free aptamers. Additionally, the intermediate Ni layer provides a magnetic response to external fields, ensuring precise control of the microrobots. Fumonisin B1 (FB1) determination can be detected with a limit of detection of 0.70 ng mL^−1^, and the system achieves concurrent FB1 and ochratoxin A (OTA) mycotoxin analysis. The “on-the-move” fluorescence quenching of the free aptamer in the rGO sensing layer also enables limits of detection for OTA and FB1 of 7 and 0.4 ng mL^−1^, respectively [52]. Functionalized QDs-based microrobots are proposed as mobile sensors for detecting food contamination [65]. Microrobot preparation involves utilizing a Pickering emulsion approach: simultaneous encapsulation of platinum nanoparticles to enhance bubble-propulsion as well as receptor-functionalized QDs for selective binding with the 3-deoxy-D-manno-oct-2-ulosonic acid, targeting the LPS from *Salmonella enterica*. The endotoxin sensing is based on the quenching of the native fluorescence of the QDs in a concentration-dependent manner (Fig. 2c). The use of microrobot-based detection reduces the detection time from several hours (the existing Gold Standard) to approximately 15 min, with the lowest detection concentration reaching 0.07 ng mL^−1^, well below the toxic level for the human body (275 μg mL^−1^). The studies discussed above demonstrate the successful application of micro/nanorobots in toxin detection and sensing, including mycotoxin, endotoxin, and ochratoxin. By integrating signal monitoring and motion control of micro/nanorobots, highly sensitive detection of raw samples can be achieved with ease.

### Gas Sensing

Toxic gas sensing strategies have been proposed using a microrobot-based sensing scheme. A polymer microrobot is constructed using a biodegradable polycaprolactone single crystal and catalase. These two components play distinct roles in sensing and self-propulsion, respectively [58]. Similar to Pt-based catalytic micro/nanorobots, propulsion is achieved through bubble recoil resulting from the catalytic decomposition of H_2_O_2_ by catalase. Utilizing the motion-induced solution mixing process and the pH-sensitive fluorescence molecule, the microrobot can effectively detect hazardous gases, such as HCl and NH_3_. Compared to its static counterpart, the dynamic microrobot demonstrates superior sensing performance, rendering it highly suitable for accurate and efficient gas detection (Fig. 2d). Taking advantage of the biodegradable nature of polycaprolactone, this microrobot has the ability to gradually degrade when immersed in a solution. Besides toxic gas sensing applications, microrobot can be applied as an oxygen sensor for intraocular measurements, which helps understand how oxygen affects various ophthalmological complications since intraocular oxygen measurements are crucial for accurate diagnosis and treatment. Ergeneman et al. propose a magnetically controlled wireless microrobot for minimally invasive intraocular oxygen concentration measurements [66]. The robot operates on the principle of luminescence quenching in the presence of oxygen, and the system integrates a luminescence optical sensor and a magnetic steering system, aiming at mobile sensing tasks at locations that are too invasive for human intervention. The microrobot can be inserted into the eye through a small incision in the sclera. Closed-loop position control within the vitreous humor can be achieved using applied magnetic fields and visual tracking through the pupil. This scheme system shows potential in mapping oxygen concentration within the eye, particularly in the preretinal area. Microrobot coated with luminescence oxygen sensor has been applied for in vitro sensing. The magnetic body enables magnetic control capability, and the coating consists of Pt (II) octaethylporphyrin (PtOEP) dyes as the luminescent material, enabling wireless excitation and optical readout [67]. Intraocular measurements can be taken based on the quenching of luminescence in the presence of oxygen.

### Biosensing

Intracellular biosensing plays an essential role in disease detection. For example, microRNAs (miRNAs) regulate important biological processes through the modulation of gene expression [68]. A nanorobot-based intracellular miRNA sensing scheme has been proposed at the single-cell level [59]. Nanorobots are designed using dye-labeled single-stranded DNA (ssDNA) coated with graphene oxide (GO) on gold nanowires. These nanorobots have the ability to penetrate intact cancer cells under the influence of ultrasound fields (Fig. 2e). The ultrasound actuation is achieved based on the concave end of the nanowire, where the scattering of ultrasound waves creates a pressure gradient and causes the nanorobot’s propulsion. Upon internalization of the nanorobot into the cell, the initially quenched fluorescence signal, resulting from the π–π interaction between GO and a dye-labeled ssDNA, is restored. This recovery is attributed to the displacement of the dye-ssDNA probe from the GO-quenching surface when it binds to the target miRNA-21, triggering the intracellular on–off fluorescence switching phenomenon. This miRNA sensing scheme is validated in MCF-7 and HeLa cancer cells, in which the fluorescence signals can be measured in real-time, demonstrating real-time intracellular miRNA expression monitoring. It is noteworthy that the biocompatibility of materials is critical for cellular sensing. Potential materials for intracellular biosensing include nucleic acid materials [69], graphene hybrid nanomaterials [70], and two-dimensional materials [71, 72].

Enzymatic glucose sensing is achieved by using bipolar electrochemistry-driven electrochemiluminescent (ECL) microrobot [73]. The propulsion (chemomechanical motion) mechanism operates by generating hydrogen bubbles at the cathodic end of the bipolar electrodes, which are directed toward the feeder anode. Simultaneous oxidation of the luminophore and enzymatically produced NADH results in ECL emission, which shows a direct correlation between glucose concentration and light intensity. This enzymatic sensing strategy combines wireless propulsion with enzymatic selectivity, demonstrating that the ECL-based readout method can be applied to moving objects. Procalcitonin (PCT) is recognized as a specific biomarker in the early clinical diagnosis of severe infectious diseases and bacterial sepsis. It is increasingly used in clinical practice to guide antibiotic therapy decisions. A microrobot-based fluorescence immunoassay is employed for PCT determination [74]. The Pt nanoparticle and Ni layers of the microrobot contribute to the catalytic self-propulsion and magnetic guidance, respectively, and the polymeric polypyrrole (PPy) outer layer enables binding capacity of the specific antibodies (anti-PCT). This strategy holds a sensitive PCT detection (0.07 ng mL^−1^) in clinical samples obtained from very low-birth-weight infants with suspected sepsis.

### Chemical Sensing

The coupling of optical properties of QDs and motion capability of micro/nanorobots enables mobile chemical sensing that provides real-time optical visualization of the analyte recognition events. Integrating fluorescence CdTe QDs onto the surface of a self-propelled tubular microrobot enables real-time detection, as the motion of the robot accelerates the binding process between trace amounts of mercury (Hg) and the QDs [97]. Selective fluorescence quenching by Hg enables effective discrimination between different mercury species and other co-existing ions, offering enhanced detection capabilities. Ag-C_3_N_4_ microrobots decompose H_2_O_2_ into oxygen bubbles under visible light irradiation, offering light-controllable motion capabilities [98]. As a metal-free microrobot, the inherent fluorescence and adsorptive capability are visualized because of the translucent body. Interestingly, the swimming capabilities of the microrobots are enhanced when exposed to prevalent toxic pollutants commonly found in wastewater, such as Pb^2+^, Cd^2+^, and Cr^2+^. This is mainly caused by the binding interactions on the robot’s surface since transition metal ions can potentially facilitate fuel decomposition. Moreover, this property is utilized to effectively remove heavy metal from contaminated water while simultaneously monitoring its adsorption through fluorescence quenching. The fluorescence quenching mechanism has also been applied for explosive detection. A fluorescent self-propelled covalent-organic-frameworks (COFs) microrobot is proposed for nitro explosive detection [75]. These microrobots can autonomously move in aqueous solutions under magnetic guidance, utilizing oxygen bubbles generated through the catalytic decomposition of H_2_O_2_ as the propulsive force. The robot exhibits fluorescence quenching in several minutes when the functionalized Py-Azine COF interacting 2,4,6-trinitrophenol (TNP) through hydrogen bond formation, in which the moving robot-induced mixing increases the detection efficiency. A Janus upconverting nanoparticle (UCNP)-functionalized polyelectrolyte microrobot displays on–off luminescence when contacting 2,4,6-trinitrotoluene (TNT) [99]. The amino groups of the poly (acrylic acid) chains on the UCNPs recognize the TNT molecules and form a Meisenheimer complex. The robot’s luminescence intensity is reduced because the fluorescence resonance energy transfer from the excited UCNPs to the complex. The limit of detection reaches 2.4 ng mL^−1^ because the collision probability between the mobile microrobot and TNT molecules is increased, and the detection can be conducted within 1 min.

Biocatalytic micro/nanorobots exhibit self-propulsion through enzymatic reactions, which can be utilized for real-time environment monitoring. The surface of mesoporous silica-based microrobots is modified with FRET-labeled triplex DNA nanoswitch for pH sensing [60]. This urease-powered microrobot can sense pH changes through FRET imaging while in motion. Its self-propulsion is achieved by urease converting urea into ammonia and carbon dioxide (Fig. 2f). During self-propulsion, the decomposition of urea and the subsequent release of ammonia led to a rapid increase in pH. This pH change is monitored in real time by assessing the FRET efficiency through confocal laser scanning microscopy. This sensing scheme enables rapid and quantitative pH detection in a matter of microseconds. A self-propelled laser microrobot is designed for the detection of extracellular vesicles and binding dynamics in complex biological fluids [100]. The microlasers are fabricated by micellar solubilization of liquid crystal, and the sensing functionality is obtained by tailoring with antibodies to capture specific proteins on extracellular vesicles. Nile red-labeled extracellular vesicles served as the gain medium to report binding events on the surface of the microcavity. When binding events take place, both radiative and nonradiative energy transfer occurs at the interface of the cavity, resulting in a shift of laser emissions from green to red bands. By monitoring the spectrally integrated laser intensities influenced by interfacial cavity energy transfer, the analysis of exosomes and biomarkers can be performed. Table 1 summarizes the recent progress on self-generated signal-based sensing strategies according to the types of micro/nanorobots, actuation methods, sensing targets, and mechanisms.

**Table 1:** Representative micro/nanorobot self-generated signal-based sensing and behavior-based sensing

System type	Micro/nanorobots	Actuation	Sensing targets	Sensing mechanism	References
Self-generated signal-based sensing	rGO/Pt microrobots	Chemically driven	Ricin B toxin	Fluorescence quenching	[61]
	WS_2_/Pt MoS_2_/Pt microrobots	Chemically driven	*E. coli* endotoxin	On–off fluorescence	[56]
	Magnetic spore-based microrobots	Magnetic field-driven	*C. diff* toxins	Fluorescence changes	[57]
	rGO/Pt microrobots	Chemical-magnetic hybrid	Fumonisin B1	Fluorescence quenching	[52]
	Polymer microrobots	Chemically driven	HCl, NH_3_	pH sensitive fluorescence molecule	[58]
	PtOEP-coated CoNi microrobots	Magnetic field-driven	Intraocular oxygen concentration	Luminescence quenching	[67]
	ssDNA/GO-coated gold nanowires	Ultrasound waves	miRNAs	Fluorescence quenching	[59]
	Covalent-organic-frameworks microrobots	Chemical-magnetic hybrid	Nitro explosive	Fluorescence quenching	[75]
	DNA microrobots	Chemically driven	pH sensing	FRET-labeled triplex DNA nanoswitch	[60]
Behavior-based sensing	Au-Pt nanorobots	Chemically driven	Ag ions and nucleic acid	Ag ions induced accelerating	[76–78]
	PEDOT-Au microtubes	Chemically driven	DNA	DNA hybridization induced accelerating	[79]
	Pt nanoparticles	Chemically driven	Zika virus	Antibody-receptor interaction induced accelerating	[80]
	Cartridge-case-like microrobot	Chemically driven	pH sensing	pH increasing induced accelerating	[81]
	Fish-like microrobot	Marangoni effect-driven	Glucose	Glucose molecules induced accelerating	[82]
	Enzyme-powered microtubes	Chemically driven	Contaminants and chemical vapor	Biocatalytic activity inhibition	[83, 84]
	Pt-based micro/nanorobots	Chemically driven	Heavy metal ions	Catalytic activity reduction	[85, 86]
	Catalases-based microbots	Chemically driven	DNA	Catalases release by DNA hybridization	[87–89]
	Pt microrobots	Chemically driven	HIV-1 RNA	DNA amplicons induced decelerating	[90]
	Pt microtubes	Chemically driven	Viscosity	Velocity varies with viscosity	[91]
	Au nanorods	Optical tweezers-driven	Viscosity	Viscosity-dependent rotating frequency	[92, 93]
	Helical microrobots	Magnetic field-driven	Flow viscosity and velocity	Environment-influenced motion	[94, 95]
	Ferrofluid droplets	Magnetic field-driven	Mechanical properties	Interact with environments through deformation	[55]
	Fe_3_O_4_ nanoparticle swarm	Magnetic field-driven	Viscosity and ionic strength	Interact with environments through deformation	[96]

## Behavior-Based Sensing

Behaviors of micro/nanorobots change when subjected to external influences (*e.g.*, changes in applied fields and surrounding environments), which can be directly observed by simple methods like optical microscopy. The behavior change caused specifically by objects that need to be detected can serve as visual signals for sensing. Behavior-based sensing of micro/nanorobots is generally achieved by establishing a quantitative relationship between the analyte and a specific micro/nanorobots behavior characteristic, and the desired information is obtained by observing the micro/nanorobots’ behaviors, such as motion and deformation.

### Accelerating Motion-Based Sensing

**Fig. 3:**
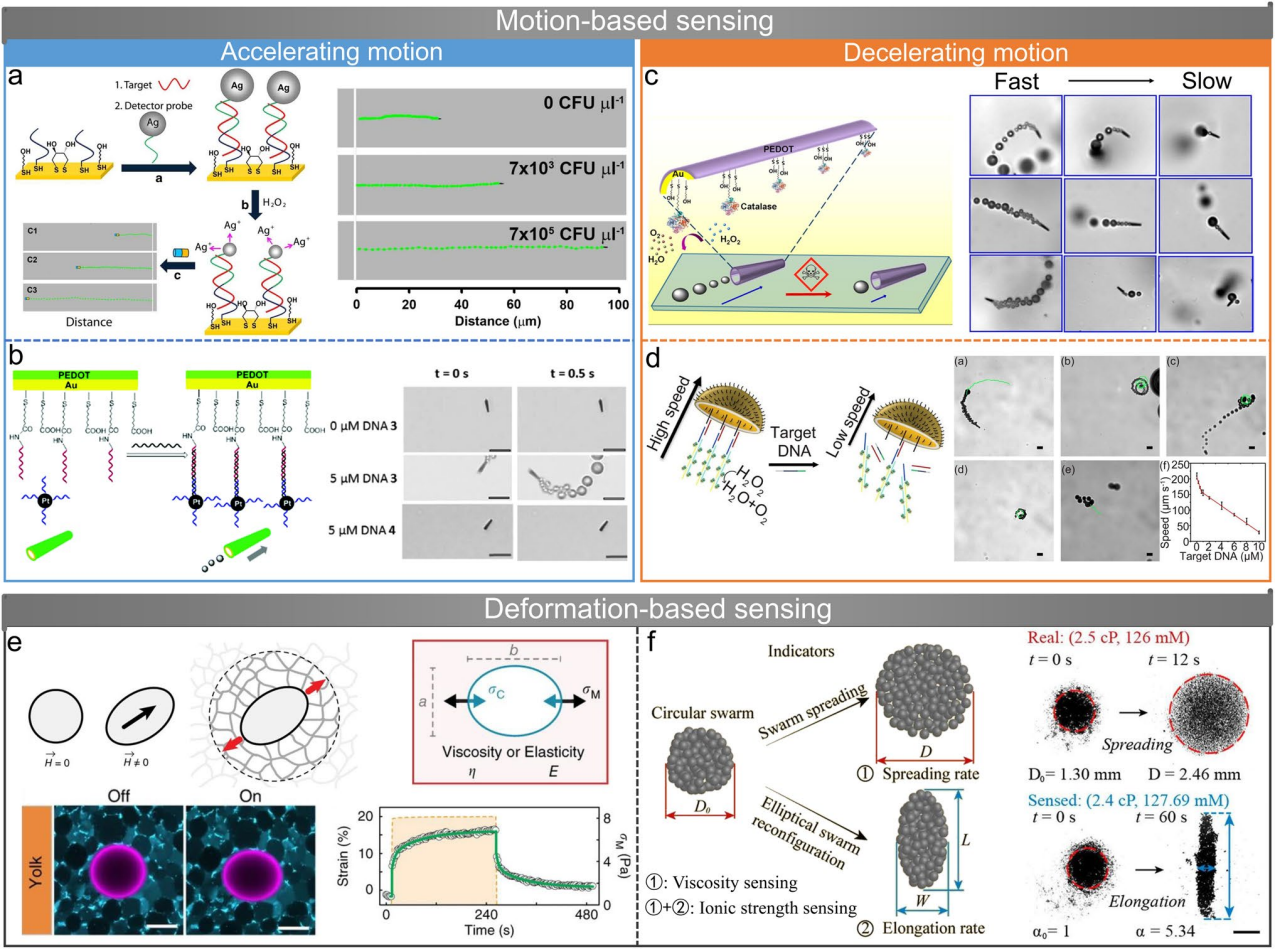
Micro/nanorobot’s behavior-based sensing. **a** DNA and RNA sensing based on the Ag nanoparticle-induced nanorobot acceleration [78]. Copyright (2010) Springer Nature. **b** The catalyst-modified PEDOT-Au microtube as a DNA sensor through specific DNA hybridization [79]. Copyright (2015) Royal Society of Chemistry. **c** Enzyme-powered microtubes used for water quality sensing based on inhibition of the catalase biocatalytic [83]. Copyright (2013) American Chemical Society. **d** Chemically powered jellyfish-like microrobot for motion-based DNA sensing [89]. Copyright (2019) American Chemical Society. **e** Local mechanical properties measurement of developing tissues and organs based on deformable ferrofluid droplets [55]. Copyright (2016) Springer Nature. **f** Pattern transformation of microrobot swarms used for sensing local fluidic viscosity and ionic strength [96]. Copyright (2022) American Chemical Society

The motion of micro/nanorobots is directly or indirectly influenced by analytes, leading to either an increase or decrease in speed. This phenomenon can be readily detected for motion-based sensing [101]. Wang et al. first report a chemical sensing platform based on the motion of fuel-driven nanorobots in 2009 [76]. They report that the velocity of Au-Pt catalytic nanorobots in H_2_O_2_ solution increases significantly in the presence of silver ions, while most other cations (*e.g.*, K^+^, Pd^2+^, and Mn^2+^) cause speed reduction. The velocity of nanorobots is positively correlated with the concentration of silver ions, so this selective acceleration phenomenon is applied to trace measurements of silver ions [76] and the location of silver source [77]. Based on the same mechanism, they further develop a motion-based specific DNA and RNA sensing platform using catalytic nanorobots [78]. As shown in Fig. 3a, silver nanoparticle-tagged detector probes (SH-DP-Ag NPs) are captured on the gold electrode due to duplex formation of complementary nucleic acid target, and the concentration of the nucleic acid target is proportional to the amount of Ag nanoparticles captured. The silver ions are released into the H_2_O_2_ solution from the Ag nanoparticle tags, increasing the speed of catalytic nanorobots in the solution. Therefore, the motion signal of nanorobots observed by optical microscopy is connected with the target nucleic acid concentration, enabling DNA detection down to the attomole level as well as direct detection of raw bacterial ribosomal RNA. Micro/nanorobots can acquire their motion capability by specifically binding with external power sources or units, and their motion behaviors serve as signals for specific sensing. Minteer et al. design a motion-based DNA sensor with DNA-functionalized Pt nanoparticles as catalysts [79]. The first type of DNA (DNA 1) is attached to a PEDOT-Au microtube, and another type of DNA (DNA 2) is conjugated to Pt nanoparticles as catalysts for the microtube, as shown in Fig. 3b. Only in the presence of the DNA target (DNA 2) will the catalyst bind to the microtube by specific DNA hybridization. The catalyst-modified microtube exhibits movement in the H_2_O_2_ solution, and the velocity increases with the concentration of DNA 3. Shafiee et al. fabricate Pt nanoparticles and polystyrene microbeads conjugated with anti-Zika virus monoclonal antibody [80]. Pt nanoparticles accumulate on the surface of the bead when Zika virus is present in the sample, resulting in the movement of the bead. The moving velocity of the microbead is positively correlated with virus concentration.

Some micro/nanorobots have pH-dependent motion behavior and thus can serve as pH sensors. Dong and colleagues report two pH sensing methods based on the motion of micro/nanorobots [81, 102]. The first is a cartridge-case-like microrobot with a gelatin shell and inner Pt nanoparticles [81]. The variation in pH value affects the volume of gelatin, the catalytic activity of Pt nanoparticles, and the volumetric decomposition of H_2_O_2_, resulting in a change in the speed of the microrobot. Another pH sensor they designed is a metal-free polymer-based microrobot consisting of polycaprolactone (PCL) and sodium 1-dodecanesulfonate (DSS) [102]. Since the structure of the microrobot is asymmetric and particles on its surfaces are unevenly distributed, the release of DSS will cause asymmetric surface tension. The side that releases more DSS has lower surface tension, causing the microrobot to move toward the high surface tension side (Marangoni effect). The release of surfactant (DSS) is affected by the pH conditions, changing the speed of the microrobot. Both microrobots move faster with larger pH values over the entire pH range and can therefore be used as pH sensors. It is worth noting that microrobot speed is negatively correlated with hydrogen ion concentration. Based on a similar principle, they also design a fish-like microrobot composed of hydrogel and surfactant [82]. Glucose molecules cause the hydrogel to swell and release the surfactant, leading to the motion of microrobots on the water surface at speed proportional to the glucose concentration. Therefore, this enzymeless microrobot can be applied to glucose sensing in human serum or urine, which may serve as an alternative to the common enzyme-based glucose detection methods.

### Decelerating Motion-Based Sensing

The presence of sensing objects may also impede the motion of micro/nanorobots, leading to a negative correlation between their motion speed and the concentration of these sensing objects. The biocatalytic activity of enzymes is susceptible to inhibition by toxins, resulting in weakened signals. This principle has been employed in the development of enzyme-based inhibition assays [103]. Similarly, the motion of enzyme-based micro/nanobots is impaired by specific agents. An enzyme-powered microtube is developed to assess the water quality based on this phenomenon [83]. As shown in Fig. 3c, the inner gold surface of the microtube is bonded with catalase, which decomposes H_2_O_2_ to generate bubbles for propulsion. The biocatalytic activity of catalase is inhibited by contaminants in water (*e.g.*, heavy metals, pesticides, and herbicides), reducing the generation rate of bubbles. The water-quality testing platform is established according to the quantitative analysis of adverse effects of various toxins on the speed and survival time of microtubes. This type of microtube is employed for remote detection of chemical vapor threats based on the inhibition of biocatalytic catalase activity by the dissolution of chemical vapor in water inhibits [84]. Considering the challenges in pinpointing the precise cause of motion reduction in enzyme-based micro/nanorobots, this sensing approach is designed to detect a broader category of substances, including water pollutants and chemical vapors, rather than focusing on a specific object. Bubble-propelled Pt catalytic micro/nanorobots have low efficiency in generating bubbles due to the absorption of heavy metal ions that cover the active site. As a result, their motion behaviors have been utilized to build sensing platforms for heavy metal ions in water. Pumera et al. find that the reduction in the movement speed of Pt microrobots due to Pb^2+^ is more significant than that caused by Cd^2+^, enabling selective detection of Pb^2+^ over Cd^2+^ in water [85]. They further explore the negative impact of various heavy metal ions on Pt-Halloysite nanorobots with more available active sites [86]. Their findings reveal that larger ions (Hg^2+^ and Pb^2+^) have a greater influence on the nanorobots’ motion due to better platinum adsorption compared to smaller ions (Zn^2+^ and Cd^2+^). The Pt-Halloysite nanorobots are employed to selectively detect and remove heavy metal ions in water solutions, following the sequence: Hg^2+^  > Pb^2+^  > Zn^2+^  > Cd^2+^. While Pt-based micro/nanorobots enable the selective detection of heavy metal ions with a low limit of detection, it may be challenging to ascertain the type and concentration of ions when multiple ions across various concentration ranges are present in the solution. One possible solution involves employing two or more types of micro/nanorobots for collaborative sensing to improve sensitivity and accuracy.

Enzyme-actuated micro/nanorobots have also been applied to DNA sensing. Wu and colleagues synthesize a microtube with catalases on its inner surface through DNA conjugate, which exhibits a significant speed reduction in the presence of target DNA [87]. Unlike sensing methods that decrease the movement of micro/nanorobots by reducing the catalytic activity of enzymes [83], DNA sensing here is achieved by reducing the amount of enzyme assembled on the microtube. Target DNA releases catalases from microtubes by DNA strand-replacement hybridization, resulting in slower self-propelled motion. They improve the DNA sensing sensitivity by constructing multiple DNA layers on the inner surface of microtubes with a cyclic alternate hybridization assembly method [88]. Each power unit contains a higher amount of catalase than before, and thus the microtube exhibits efficient propulsion even in low-concentration H_2_O_2_ solutions. When the multilayer DNA and catalases are released due to hybridization with target DNA, decreases in microtube velocity are more easily observed. In further work, they improve the structure of the DNA sensor and design a jellyfish-like microrobot consisting of a multimetallic shell and DNA architecture with catalase on the concave surface (Fig. 3d) [89]. Although the detection principle is still displacement hybridization-triggered catalase release, the jellyfish-like microrobot has higher sensing sensitivity because the open surface allows the target DNA to bind better to the sensing unit than the tubular surface. It is reported in another study that DNA-engineered Pt microrobots are used for HIV-1 RNA detection [90]. Large-sized DNA amplicons are formed from HIV-1 through loop-mediated isothermal amplification reaction and then hybridized into DNA probes on the microrobots. Unlike their proposed Zika virus sensing method [80], DNA amplicons captured by microrobots provide no additional power but rather act as a burden that hinders motion. The large DNA tail results in an obvious velocity reduction of the microrobot, which establishes the connection between the motion of Pt microrobots and HIV-1 RNA concentrations. A shared characteristic of these sensing methods is that they establish a connection between the concentration of target nucleic acid molecules and the motion speed of micro/nanorobots through DNA hybridization, yielding excellent specificity and sensitivity. This design concept is expected to be extended to micro/nanorobots driven by various methods, providing a straightforward visualization approach for DNA/RNA sensing.

Surrounding environments have significant influences on the motion behaviors of micro/nanorobots. Therefore, in addition to specific substances such as ions and DNA molecules, motion behaviors of micro/nanorobots are also used to detect the physical properties of their surrounding environments. Generally, it is more difficult for micro/nanorobots to move in fluids with higher viscosity due to smaller Reynolds numbers. Zhang et al. investigate the motion behavior of Pt microtubes in H_2_O_2_ solutions with different viscosities and establish a hydrodynamic model [91]. The microtubes are used as viscometers due to the negative linear correlation between their motion speed and fluid viscosity. The optical tweezers-driven rotation of micro/nanorobots is influenced by the viscosity of surrounding fluids. Käll et al. report the extremely fast rotation of gold nanorods in aqueous solutions through optical torques and find that the rotating frequency decreases with increasing viscosity [92]. The optical tweezer generates a microvortex by driving SiO_2_ particles or yeast cells along a fixed circular trajectory, indirectly rotating cells within the trajectory [93]. The rotation of micro/nanorobots results from viscous stress and has a frequency that is negatively correlated with ambient fluid viscosity. Ghosh et al. estimate fluid viscosity by observing the position and orientation of a helical microrobot driven by external magnetic fields [94]. They treat the helical microrobot as a ferromagnetic rod and establish a relationship among the critical frequency (${\Omega }_{1}$), the rotating frequency of the magnetic field (${\Omega }_{B}$), and the precession angle (${\alpha }_{p}$) as ${\alpha }_{p}={\text{sin}}^{-1}\left({\Omega }_{1}/{\Omega }_{B}\right)$. Therefore, the critical frequency can be obtained by measuring the precession angle, and then, the viscosity of the local fluid is calculated. This method is validated in both Newtonian and shear-thinning fluids, allowing viscosity measurements in the range of 100 cP. Micro/nanorobots designed for liquid viscosity sensing require a stable motion state and motion behavior correlated with viscosity, which are crucial factors for obtaining accurate viscosity measurements. The small size of micro/nanorobots makes them particularly well-suited for measuring the viscosity of minute liquid samples, such as viscosity sensing in micro/nanoelectromechanical systems or even in vivo environments. In addition, microrobots moving in dynamic environments can also reflect flow information. The local flow velocity is obtained by comparing the actual velocity of a magnetic helical microrobot in flow with its theoretical velocity in the absence of flow, benefiting the optimization of motion control [95].

### Deformation-Based Sensing

The magnetic field can not only drive the motion of micro/nanorobots but also induce their deformation behavior, which is influenced by the surrounding environment [104]. Campàs et al. propose a strategy to directly measure the local mechanical properties using deformation of micro/nanorobots, with biocompatible magnetic ferrofluid microdroplets as sensors (Fig. 3e) [55]. The ferrofluid droplet can elongate into an ellipsoid under a uniform magnetic field with its major axis along the field direction [105]. The strain of the droplet is related to the mechanical properties (viscous or elastic elements) of the surrounding materials. They establish a 1D description of the complex mechanical properties of surrounding materials and droplet deformation. The feasibility of using ferrofluid droplets as micro-rheometers and micro-tensiometers is validated in materials with known properties. Finally, in vivo quantitative spatiotemporal measurements of tissue-level mechanical properties in developing zebrafish embryos are demonstrated. Yu and colleagues develop a vortex-like nanoparticle swarm with reversible pattern reconfiguration ability [106] and use the deformation behavior of the microrobot swarm to detect local fluidic viscosity and ionic strength [96]. The influence of fluidic properties on the spreading and elongation of the vortex-like swarm is investigated, and data models for sensing fluidic viscosity and ionic strength are established. By measuring the rates of spreading and elongation, the viscosity and ionic strength of porcine whole blood are successfully measured (Fig. 3f). Furthermore, the deformation of microrobots has also been used for force sensing, which is achieved by observing the degree of deformation of elastic structures in microrobotic systems [107, 108]. The methods enable the measurement of forces at the micro-Newton level or lower, *e.g.*, forces for manipulating biological cells.

In addition to behaviors like locomotion, rotation, and deformation, the tactic behavior of micro/nanorobots holds potential for the development of intelligent sensing platforms. Here, tactic behavior refers to micro/nanorobots behaving like many biological organisms and moving toward or away from certain signal sources, such as chemical gradient, light source, gravity, flow, and magnetic field [109, 110]. For instance, chemically driven micro/nanorobots exhibit chemotaxis in response to varying fuel concentrations [111, 112]. It has been reported that platinum-gold microrods spontaneously move toward areas with higher H_2_O_2_ concentrations when there is a gradient present [113]. A polymer microsphere with catalytic palladium randomly distributed on its surface shows tactic behavior in H_2_O_2_ solutions with a pH gradient, which is attributed to the solute pressure imbalance across its surface [114]. The microsphere can perform specific behaviors, including random walking, translation, vertical movement, jumping, and pulsating movement, when adjusting the pH gradient or its size. Mou et al. develop a ZnO-based Janus microrobot powered by the biocompatible fuel CO_2_, which displays intelligent positive chemotaxis to CO_2_ gradient [115]. The propulsion is a result of the electrolyte self-diffusiophoresis induced by the CO_2_ dissolution-caused ZnO corrosion, while the tactic behavior arises from the phoretic torque generated by the unbalanced electro-osmotic slips when the microrobot’s axis is misaligned with the chemical gradient. These tactic behaviors of micro/nanorobots can be employed to sense information about their surroundings, such as local gradients of H_2_O_2_, pH, and CO_2_. Consequently, developing intelligent micro/nanorobots with tactic behavior as sensors in complex and variable environments signifies a promising avenue for building micro/nanorobot-based intelligent sensing platforms. In conclusion, Table 1 provides a summary of representative sensing strategies based on behaviors of micro/nanorobots.

## Micro/Nanorobots Selectively Capture and Transport Targets for Sensing

The isolation of target analytes from raw samples is a necessary preliminary step in many common sensing methods, often requiring time-consuming and intricate procedures. Micro/nanorobots are emerging as alternative tools for effectively isolating target analytes, thereby enhancing the efficiency of sample pretreatment in sensing. The isolation process based on micro/nanorobots typically involves two steps: capture and transportation. Micro/nanorobots utilize specific reactions to recognize and capture target analytes, which are then transported to the desired location through active motion. This allows for the isolation of target analytes from complex raw samples, enabling precise and quantitative sensing. In subsequent sensing processes, external stimuli can be applied to trigger the release of the captured analytes. With their small size and controllable active motion, micro/nanorobots demonstrate exceptional capability in isolating targets from intricate samples, especially when dealing with small volumes and low concentrations. This section presents research studies in which micro/nanorobots are utilized to selectively capture, transport (and release) targets for sensing tasks.

### Biological Motor-Based Capture

**Fig. 4:**
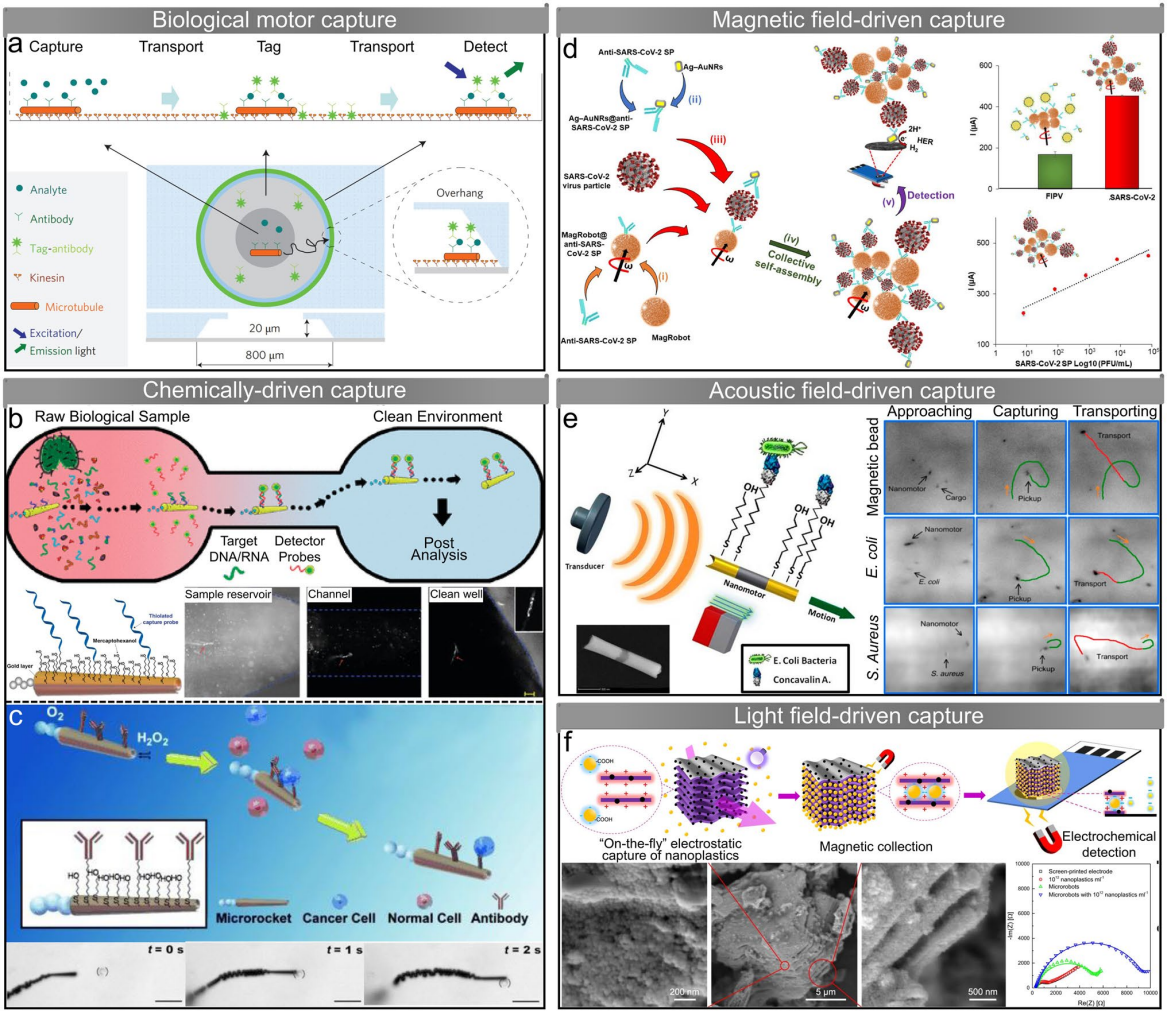
Micro/nanorobots selectively capture and transport targets for sensing. **a** A “smart dust” microdevice based on biomolecular motors for capturing and transporting target analytes [119]. Copyright (2009) Springer Nature. **b** Bubble-propelled catalytic Ti/Ni/Au/Pt microtubes for isolating nucleic acid from raw biological samples. The optical images show the microtube capturing target DNA from a sample reservoir and transporting it through a 6 mm-long channel into a clear well [122]. Copyright (2011) American Chemical Society. **c** The Ti/Fe/Au/Pt microtubes selectively capture target cancer cells through specific antigen recognition. The optical images show the microtube capturing and transporting a CEA+ pancreatic cancer cell in diluted human serum [123]. Copyright (2011) John Wiley and Sons. **d** Magnetic microrobots capture SARS-CoV-2 virus and Ag-AuNR tags and self-assemble into 3D-staggered chains through immuno-sandwich assay. Subsequent detection is achieved based on the hydrogen evolution reaction of Ag-AuNR tags [124]. Copyright (2022) Elsevier. **e** Ultrasound-powered magnetically guided Au-Ni-Au nanowires for selective capture and transport of bacteria. The optical images show capture and transport of a magnetic bead, *E. coli* bacteria, and *S. aureus* bacteria, respectively [125]. Copyright (2013) American Chemical Society. **f** Light-powered MXene-derived γ-Fe_2_O_3_/Pt/TiO_2_ microrobots for capture and detection of nanoplastics. The SEM images show microrobots with nanoplastics captured [126]. Copyright (2022) Springer Nature

Motor proteins (such as kinesin and myosin) consume energy from adenosine triphosphate (ATP) hydrolysis to move along the cytoplasm of cells and transport specific substances (*e.g.*, proteins and vesicles) [116]. The unique mechanism enables micro/nanorobots actuated by biomolecular motors to serve as carriers for capturing and transporting target analytes in sensing tasks [117, 118]. In a “smart dust” sensing microdevice, the wash steps in traditional double-antibody sandwich assays are replaced by transport steps based on molecular shuttle (Fig. 4a) [119]. The target analytes (streptavidin or glutathione-S-transferase) are captured by kinesin-powered antibody-functionalized microtubules and transported to bind fluorescent markers, and the microtubules loaded with both analytes and markers move finally into the detection region. DNA hybridization technique is incorporated into biomolecular motor transport-based sensing systems to achieve target loading and unloading [120, 121]. The microtubules and cargo modified with complementary DNA bind to each other at specific sites by DNA hybridization, and the cargo is subsequently unloaded at the location where the DNA complementary to that labeled on the cargo is attached.

### Chemically Catalytic Micro/Nanorobots-Based Capture

The limited lifespan and isolating efficiency of biological motors in complex raw samples limit their application in sensing tasks. Taking advantage of their excellent motion ability, ease of functionalization, and wide actuation environments, synthetic micro/nanorobots have become a promising option for capturing and transporting target analytes [127]. Among them, chemically catalytic micro/nanorobots, especially tubular micro/nanorobots (microtubes), have been widely reported for the isolation of nucleic acids, proteins, cells, bacteria, etc. [128]. Spherical mesoporous silica particles functionalized with catalase on one side and single-stranded DNA on another side are fabricated for DNA capture [129]. The catalase catalyzes the breakdown of H_2_O_2_ to generate bubbles for actuation of the asymmetrically functionalized microspheres, while the modified DNA allows rapid isolation and subsequent detection of the target oligonucleotide sequence. Wang and colleagues fabricate bubble-propelled catalytic Ti/Ni/Au/Pt microtubes for isolating nucleic acid targets from a variety of untreated biological samples (*e.g.*, serum, urine, and saliva), as shown in Fig. 4b [122]. The external gold layer of the microtube is modified with single-strand DNA, which binds with the target nucleic acid through DNA hybridization. These microtubes are able to transport the captured nucleic acid to a clean area for subsequent analysis.

Bubble-propelled microtubes have been reported to isolate target proteins from complex raw biological samples [130]. The Ti/Ni/Au/Pt microtube is functionalized with thiolated thrombin aptamer (SH-TBA) onto its outer gold surface, endowing the microtube with the ability to capture and transport the target thrombin protein. To achieve controlled release of the captured thrombin protein, the TBA receptor is replaced by a mixed binding aptamer (MBA) receptor, followed by the addition of ATP. The added ATP can bind and replace the immobilized MBA aptamer, triggering the release of captured thrombin. Molecularly imprinted polymers (MIP) are used to synthesize microtubes capable of selectively transporting proteins [131]. The electropolymerization of the microtube outer polymeric layer is carried out in the presence of the target protein (avidin), and the obtained microtube surface has selective recognition sites of the target protein through complementary nanocavities. After the electrodeposition of Pt and Ni in the inner layer, MIP-based microtubes with abilities to recognize, capture, and directional transport target proteins without functionalization of additional receptors are obtained. The MIP-based micro/nanorobots can be extended to the selective transport of various target analytes. Antibody-modified Fe_3_O_4_@ SiO_2_/Pt nanoparticles are fabricated to cruise and capture immunoglobulin (IgG) via self-propelled motion [132]. Core–shell Au@Ag nanocubes that serve as labels of secondary antibodies bind with captured IgG to amplify electrochemical signals. The sandwich immune-complex is formed through self-assembly and can transfer rapidly on the electrode under magnetic actuation, enabling the sensing of IgG by using differential pulse voltammetry. Micro/nanorobots can capture target proteins and serve as labels for electrochemical detection [133]. Self-propelled IrO_2_/Pt bilayer microtube and tosyl-activated magnetic beads are modified with anti-rabbit IgG, and they are coupled in the presence of target rabbit IgG. The self-propelled motion of the microtube facilitates the capture, while the magnetic beads make the immune sandwich assay easy to separate and fix on the electrode. The electrocatalytically active IrO_2_ outer layer can enhance hydrogen evolution reaction for electrochemical detection of target proteins. A rapid on-the-fly C-reactive protein (CRP) sensing method is developed based on magnetic graphene microtubes [134]. The microtube has an outer rGO layer, a middle Ni layer, and an inner Pt nanoparticles layer, which are responsible for antibody functionalization, magnetic guidance, and propulsion, respectively. The rGO layer is first immobilized with streptavidin and then modified with CRP antibody. The antibody-modified microtubes are actuated in solutions containing the CRP targets and anti-CRP detection antibody labeled with horseradish peroxidase (HRP) to form the sandwich immunocomplex for electrochemical measurements. This method allows rapid (5 min) and accurate determination of CRP in small-volume plasma samples (< 10 *μ*L). Ma et al. fabricate microtubes with an outer layer of mesoporous silica (mSiO_2_) and an inner layer of TiO_2_ modified with Fe_3_O_4_ and Pt nanoparticles [135]. The presence of the outer mSiO_2_ layer endows the TiO_2_@mSiO_2_ microtube with water pollutants adsorption ability three times higher than that of common TiO_2_ microtubes. Microtubes that absorb pollutants can be collected by magnetic attraction and then placed in deionized water to release their absorbates. The composition of pollutants is further analyzed for environmental monitoring.

Larger targets, such as cells and bacteria, can also be selectively captured and transported by micro/nanorobots. The Ti/Fe/Au/Pt microtube is modified with anti-carcinoembryonic antigen (anti-CEA) monoclonal antibody on its external gold surface [123], where the CEA is one of the most common antigens among cancer cells [136]. The antibody-modified microtubes are able to selectively capture target cancer cells through specific antigen recognition in PBS and serum (Fig. 4c). The microtubes can generate enough force (> 13 pN) to overcome the additional resistance after capturing the cells and perform continuous directional transport under the guidance of the magnetic field. Although the chemical fuel (hydrogen peroxide) required for the propulsion of the microtubes affects the viability of the cells, the low H_2_O_2_ concentration (2%) allows most isolated cells to survive for a certain period of time (1 h) for subsequent analysis. Besides, even dead target cells or fragments of their cellular membrane can be identified and captured by the microtube. Another boronic acid-based microtube demonstrates the ability to capture yeast cells through selective monosaccharide recognition [137]. The outer layer of the polymer/Ni/Pt microtube is poly (3-aminophenyl boronic acid) (PAPBA), which can form complex with monosaccharides and is widely used for glucose sensing [138, 139]. The PAPBA outer layer endows the microtube with “built-in” monosaccharide recognition ability without the need for additional functionalization and has no significant effect on the catalytic propulsion. Yeast cells with sugar residues on their walls are successfully captured and transported by the microtubes. The release of captured yeast cells can be triggered by the addition of fructose, which has a stronger affinity for boronic acid. A lectin receptor-functionalized microtube has been reported for the capture, transport, and release of *E. coli* [140]. Concanavalin A (ConA) lectin bioreceptor modified on the surface of Au/Ni/polyaniline/Pt microtube can selectively recognize and bind carbohydrate constituents on the bacterial surface (such as polysaccharides on the surface of *E. coli*) [141], enabling the microtube to capture *E. coli* in various complex real samples (*e.g.*, drinking water, apple juice, and seawater). The release of captured *E. coli* is achieved by dissociating the lectin-bacteria complex using a low-pH glycine solution.

### External Field-Driven Micro/Nanorobots-Based Capture

While the capture and transport abilities of typical catalytic micro/nanorobots for various targets have been demonstrated in numerous studies, their reliance on additional chemical fuels can restrict their application in sensing scenarios involving analytes sensitive to such fuels. In addition to chemically driven micro/nanorobots, there are external field-driven micro/nanorobots, including those driven by magnetic fields, acoustic fields, and light fields, which have also been reported to possess selective capturing and transportation capabilities.

Paramagnetic nanoparticles are modified with antibody against SARS-CoV-2 spike protein to selectively capture and pre-concentrate SARS-CoV-2 virus under applied rotating magnetic fields (Fig. 4d) [124]. Silver-shell/gold-core nanorods (Ag-AuNRs) modified with antibody against SARS-CoV-2 spike protein are used as electro-catalytic labels. The nanoparticles self-assemble into 3D-staggered chains through immuno-sandwich assay instead of individual or linear chains, significantly enhancing the capture and transport abilities. Linear sweep voltammetry is conducted to measure hydrogen evolution reaction catalysis of Ag-AuNRs in the magnetic immuno-sandwich assay, and enhanced current intensity is detected in the presence of SARS-CoV-2 virus particles. Ultrasound-powered magnetically guided three-segment Au-Ni-Au nanowires exhibit the ability to isolate bacteria [125]. A sphere lithography technique is used to create concavity at the end of the nanowire to optimize the asymmetric acoustic pressure distribution, significantly improving the acoustic actuation performance (Fig. 4e). The Ni part allows the nanowire to navigate under an applied magnetic field. The surface of the gold part is functionalized with specific bioreceptors to selectively capture and transport target bacteria, such as *E. coli* and Staphylococcus aureus (*S. aureus*), in complex samples. Pumera et al. design light-powered MXene-derived γ-Fe_2_O_3_/Pt/TiO_2_ microrobots to capture and detect nanoplastics [126]. The MXene multilayered structure offers an engineered electrostatic attraction to trap nanoplastics on the slits between multilayer stacks of the microrobots [142]. The microrobots perform effective 3D negative photogravitactic motion to capture nanoplastics in the whole space under the irradiation of ultraviolet (UV) light due to Pt deposition on the flat sides of the TiO_2_ microparticles (Fig. 4f). The decoration of magnetic γ-Fe_2_O_3_ nanoparticles allows the microrobots to be collected from treated water for the preconcentration and further detection of nanoplastics by electrochemical impedance spectroscopy using low-cost and miniaturized screen-printed electrodes. Star-shaped BiVO_4_ microrobots perform self-propulsion under the irradiation of visible light in pure water due to asymmetrical photogeneration of chemical ions [143]. The local chemical gradient induced by light enables the BiVO_4_ microrobots to capture and transport surrounding objects (*e.g.*, passive particles and living microorganisms) through phoretic attraction, and the release can be achieved by turning off the light field. Moreover, the BiVO_4_ microrobots tended to move toward and capture yeast cells in the presence of other microorganisms (*e.g.*, *E. coli*) without additional surface functionalization and magnetic guidance. The selective capture is attributed to the rough surface, hydrophobicity, and intrinsic surface interactions of BiVO_4_ microrobots and yeast cells. Recently, another light-powered titanium dioxide-silica Janus particle is reported, capable of capturing and transporting passive microparticles and *E. coli* bacteria in the aqueous solution through dominant attractive van der Waals forces [144].

It is worth mentioning that micro/nanorobots as capturers perform rapid motion to increase contact with targets and significantly improve capture efficiency. The convection of surrounding fluid induced by the micro/nanorobots plays an important role in the rapid capture as well. In particular, the bubble-propelled micro/nanorobots generate bubbles to induce strong convection while providing sufficient propulsion for transporting captured cargoes. Compared with static capturer-based analytes isolating methods, active micro/nanorobots provide a promising method for faster and more efficient capture and transport of analytes in three-dimensional space for sensing. Related research works are summarized, as shown in Table 2.

**Table 2:** Representative studies on utilizing micro/nanorobots to capture and transport targets for sensing purposes and micro/nanorobot-assisted sensing

System type	Micro/nanorobots	Actuation	Sensing targets	Sensing mechanism	References
Selective capture and transport for sensing	Antibody-modified microtubules	Kinesin-powered	Protein and enzyme	Antibody-receptor interactions	[119]
	Antibody-modified microtubules	Kinesin-powered	DNA	DNA hybridization	[120, 121]
	Spherical mesoporous silica particles	Chemically driven	DNA	DNA hybridization	[129]
	Ti/Ni/Au/Pt microtubes	Chemically driven	Nucleic acid	DNA hybridization	[122]
	Ti/Ni/Au/Pt microtubes	Chemically driven	Thrombin protein	Antibody-receptor interactions	[130]
	MIP-based microtubes	Chemically driven	Avidin	Nanocavities enabled recognition	[131]
	Fe_3_O_4_@SiO_2_/Pt nanoparticles	Chemically driven	Immunoglobulin	Sandwich immunoassay	[132]
	IrO_2_/Pt microtubes	Chemically driven	Immunoglobulin	Sandwich immunoassay	[133]
	Magnetic graphene microtubes	Chemically driven	C-reactive protein	Sandwich immunoassay	[134, 145]
	TiO_2_@mSiO_2_ microtubes	Chemically driven	Pollutants	Surface absorption	[135]
	Ti/Fe/Au/Pt microtubes	Chemically driven	Cancer cells	Antibody-receptor interactions	[123]
	Boronic acid-based microtubes	Chemically driven	Yeast cells	Monosaccharide recognition	[137]
	Au/Ni/polyaniline/Pt microtubes	Chemically driven	*E. coli*	Antibody-receptor interactions	[140]
	Paramagnetic nanoparticles	Magnetic field-driven	SARS-CoV-2 virus	Antibody-receptor interactions	[124]
	Au-Ni-Au nanowires	Acoustic field-driven	Bacteria	Antibody-receptor interactions	[125]
	γ-Fe_2_O_3_/Pt/TiO_2_ microrobots	Light field-driven	Nanoplastics	Electrostatic trapping	[126]
Micro/-nanorobot-assisted sensing	Polyaniline-Pt microrobots	Chemically driven	Proteins	Enhancing mixing	[146]
	Fe_3_O_4_ nanoparticle chains	Magnetic field-driven	*E. coli* O157:H7 DNA and PSA	Enhancing mixing	[147]
	Mg/Pt Janus microrobots	Chemically driven	Glucose	Enhancing mixing	[53]
	Functional silk-based microrobots	Chemically driven and Marangoni effect-driven	Apolipoprotein E	Enhancing mixing	[148]
	Mg-Ni-Au Janus microrobots	Chemically driven	Organophosphorus nerve agents	Enhancing mixing	[149]
	Mg/Au Janus microrobots	Chemically driven	Diphenyl phthalate	Promoting degradation	[150]
	γ-Fe_2_O_3_/SiO_2_ nanoparticles	Magnetic field-driven	H_2_O_2_ and glucose	Triggering colorimetric reaction	[151]
	Enzyme-based microtubes	Marangoni effect-driven	H_2_O_2_	Triggering colorimetric reaction	[152]
	PEDOT/Ni/Pt microtubes	Chemically driven	Cortisol	Triggering colorimetric reaction	[153]
	SW-Fe_2_O_3_/MnO_2_ microrobots	Chemically driven	Phenylenediamine isomers	Triggering colorimetric reaction	[154]
	Fe_3_O_4_/Au/Ag nanoparticles	Magnetic field-driven	SARS-CoV-2 RNA	Hybridized duplex release	[54]
	Pt/Ag_3_VO_4_ Janus particles	Light field-driven	Citric acid	Ag irons release	[155]
	Magnetic nanoparticles	Magnetic field-driven	*E. coli*	Offering Raman labels	[156]
	Au/SiO/Ti/Ag microtubes	Chemically driven	Rhodamine 6G	Enrichment of analytes	[157]
	Bimetallic Au/Ag core/shell nanorods	Chemically driven	Picric acid	Enrichment of analytes	[158]
	Match-like one-dimensional nanorobots	Light field-driven	Crystal violet and cancer cells	Enrichment of analytes	[159]
	Gold nanorods	Acoustic field-driven	DNA	Enrichment of analytes	[160]
	Au/SiO/Fe microtubes	Magnetic field-driven	Rhodamine 6G	Hotspots-based SERS enhancement	[161]
	Silica-coated Fe_3_O_4_ nanoparticles	Magnetic field-driven	Dye and biomolecules	Hotspots-based SERS enhancement	[162]

## Micro/Nanorobots-Assisted Sensing

Micro/nanorobots can play an auxiliary role in conventional sensing methods, such as electrochemical sensing, colorimetric sensing, and surface-enhanced Raman scattering (SERS) sensing, to enhance the sensing performance. We classify methods in which micro/nanorobots assist sensing by exerting influence on environments, including enhancing mass transfer, triggering reactions, and accelerating reaction rates, as micro/nanorobot-assisted sensing (Table 2).

### Enhancing Mixing

As mentioned above, micro/nanorobots moving in a fluid environment inevitably affect surrounding fluids. Wang et al. quantitatively characterize the fluid convection and mixing induced by bubble-propelled microrobots using passive polystyrene microbeads as tracers [48]. The mean squared displacement (MSD) of passive tracers becomes significantly larger under the influence of bubble-propelled microrobots, providing evidence for enhanced fluid transport resulting from the motion of microrobots. The efficiency of induced mass transfer can be further enhanced through special design of micro/nanorobots. Guan et al. design a bowl-shaped Janus polystyrene/Pt microrobot featuring an axis-asymmetric hollow structure, which is able to perform unique precession, synchronistic translation, and rotation [163]. Compared with typical Janus microrobots, the “on-the-fly” mass transfer generated by the axis-asymmetric microrobot is considerably multiplied due to the unusual motion mode. The enhanced fluid convection and mixing induced by micro/nanorobots show promising potential for improving sensing efficiency, and micro/nanorobots enhanced mixing-assisted sensing methods have been developed. In these methods, micro/nanorobots do not generate sensing signals or act as carriers for analytes; rather, their active movement is solely employed to improve the effectiveness of sensing through enhancing mixing.

**Fig. 5:**
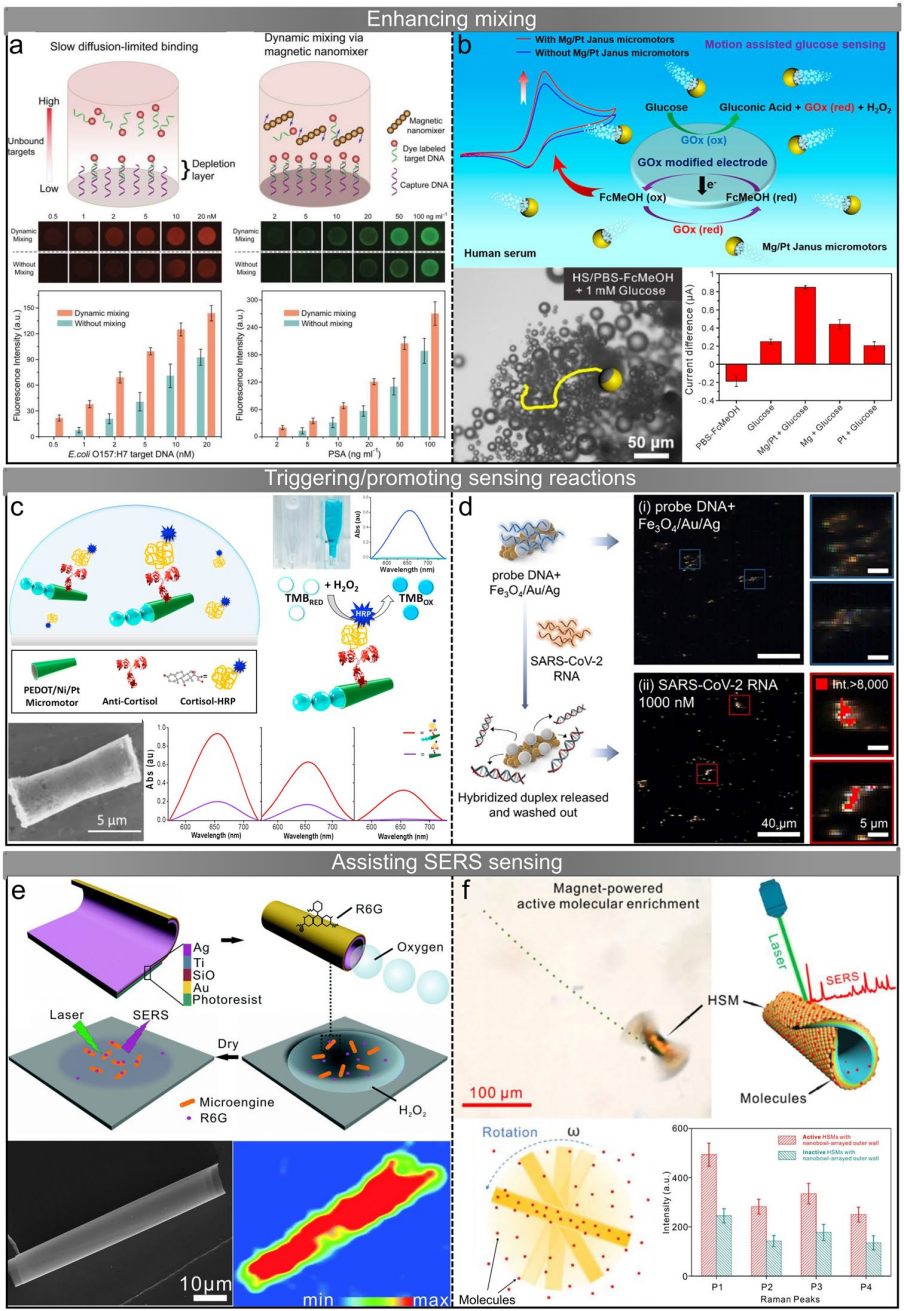
Micro/nanorobot-assisted sensing. **a** Polydopamine-crosslinked magnetic Fe_3_O_4_ nanoparticle chains enhance mixing in a microarray-based sensing system through rotating [147]. Copyright (2018) John Wiley and Sons. **b** Mg/Pt Janus microrobots accelerate oxidation of glucose for electrochemical sensing through enhancing mass transfer [53]. Copyright (2019) American Chemical Society. **c** PEDOT/Ni/Pt microtubes for rapid naked-eye cortisol sensing [153]. Copyright (2017) Elsevier. **d** Plasmonic-magnetic Fe_3_O_4_/Au/Ag nanorobots for SARS-CoV-2 RNA sensing [54]. Copyright (2022) Elsevier. **e** Au/SiO/Ti/Ag microtubes for enhanced SERS sensing through molecule enrichment. Reproduced with permission from [157]. Copyright (2016) Royal Society of Chemistry. **f** Au/SiO/Fe microrobots serve as mobile hotspots to enhance SERS signals [161]. Copyright (2020) American Chemical Society

The microarray technique is widely used in high-throughput analysis of small molecules such as DNA and proteins, however, the diffusion speed of molecules from solution to the surface of immobilized probes is relatively slow [164, 165]. Merkoçi and colleagues study the enhancement of solution mixing by self-propelled microrobots in microarray-based immunosensing [146]. Polyaniline-Pt microrobots generate bubbles in the H_2_O_2_ solution for propulsion, inducing local convection and vortex streams to promote mass transfer. The binding between molecular receptors (immobilized antibody microarrays) and target molecules (protein biomarkers) is enhanced with the assistance of microrobots, resulting in a relative increase in the detected signal intensity of up to 3.5-fold. In another study, polydopamine-crosslinked magnetic Fe_3_O_4_ nanoparticle chains are used as nanomixers to facilitate solution mixing in a microarray-based sensing system (Fig. 5a) [147]. The nanoparticle chains perform synchronous rotation under an external rotating magnetic field, generating steady circular fluid flowing around them. The movement of the molecules is significantly accelerated by the generated flow, increasing the detection sensitivity of *E. coli* O157:H7 DNA and prostate specific antigen (PSA) by more than four-fold. In addition, the enhanced mixing reduces spot-to-spot variability in the microarray system to less than 10%.

Electrochemical sensing of glucose in human serum is reported to be enhanced by microrobot-induced mixing [53]. A glucose biosensor system is developed based on the enzymatic oxidation of glucose by glucose oxidase and electrochemical detection of the mediator (FcMeOH). Mg/Pt Janus microrobots are able to generate hydrogen bubbles for self-propulsion in human serum without additional chemical fuels [166]. The rapid movement of the microrobots and bubble-induced convection enhance the mass transfer, resulting in accelerated oxidation of glucose and FcMeOH on the electrode surface (Fig. 5b). The intensity of the measured current signal is proportional to the number of Mg/Pt Janus microrobots, and the limit of detection is improved when the microrobots concentration becomes higher. Zhao et al. use reactive inkjet printing to fabricate two microrobot stirrers capable of performing autonomous rotation for enhancing mixing, where the rotation is achieved through the Marangoni effect and the catalytic bubble generation, respectively [148]. Enzyme-blended regenerated silk fibroin inks are used to print the bubble-propelled microrobots, which endows the microrobots with the ability to decompose H_2_O_2_ for propulsion. They find that more defined circular rotation can be obtained by distributing catalase at both ends of the microrobot. Based on the rapid leaching of the additive (polyethylene glycol) contained in the ink to generate a surface tension gradient, they fabricate Marangoni effect-driven microrobots by using inks without enzymes. The Marangoni effect-driven microrobots generate rotation (600 rpm) 75–100 times faster than the enzyme-driven microrobots without additional chemical fuel, but their lifetime is shorter than that of the enzyme-powered microrobots. These two microrobot stirrers are expected to be used in sensing methods that require stirring to conveniently improve the sensing efficiency.

### Triggering/Promoting Sensing Reactions

In addition to speeding up mass transfer through enhancing mixing, micro/nanorobots can trigger or promote the reactions required for sensing. In electrochemical measurements of organophosphorus (OP) nerve agents, Mg-Ni-Au Janus microrobots confined on the surface of sensor strips play the role of enhancing the amperometric signal [149]. While enhancing mass transfer through bubble generation, the microrobots also promote the degradation of non-detectable OP nerve agents (paraoxon) into electroactive compounds (p-nitrophenol) for electrochemical sensing without the need for additional reagents or instruments. The accelerated hydrolysis of paraoxon is attributed to the dramatic increase of pH by Mg-generated hydroxyl ions in the aqueous solution. Such dual-action microrobots significantly improve the sensitivity of paraoxon sensing by up to 15-fold. Based on a similar mechanism, Escarpa and colleagues design an Mg/Au Janus microrobot-based method for the sensing of organic pollutants (diphenyl phthalate, DPP) in food and biological samples [150]. On the one hand, the microrobot generated hydrogen bubbles for propulsion and enhanced mass transfer with NaCl acting as fuel and supporting electrolyte; on the other hand, the hydroxyl ions generated by Mg promote the rapid degradation of the non-electroactive DPP to electroactive phenol, which can be directly measured by differential pulse voltammetry on disposable screen-printed electrodes. This sensing method based on the dual action of Mg/Au Janus microrobots enables fast detection (~ 5 min) and high recovery (~ 100%), increasing the sensing sensitivity of DPP by about 20 times. Besides paraoxon and phenol, the increase in environmental pH induced by Mg-based micro/nanorobots is expected to be extended to the sensing and degradation of other pollutants, *e.g.*, the degradation of non-electroactive carbofuran to electroactive carbofuran phenol in alkaline solution. Moreover, micro/nanorobots based on other catalytic materials (*e.g.*, Zn and Pt) can also play a role in changing the pH of the environment while enhancing mixing.

Micro/nanorobots are able to change the color of solutions through reactions with analytes to achieve colorimetric detection. Ying et al. fabricate multifunctional asymmetric hematite-silica hybrid Janus γ-Fe_2_O_3_/SiO_2_ nanoparticles for sensitive colorimetric detection of H_2_O_2_ and glucose [151]. These magnetic Janus nanoparticles have intrinsic peroxidase-like catalytic activity and can maintain stable performance in a wider range of pH and temperature than natural enzymes. In the presence of H_2_O_2_, the chromogenic peroxidase substrate (3,3’,5,5’-tetramethylbenzidine, TMB) is oxidized to blue products by the catalyzed hydroxyl ions from H_2_O_2_ degradation, which enables the detection of H_2_O_2_ by the naked eye or the change of absorbance at 652 nm. The SiO_2_ surface of the Janus particles is modified with glucose oxidase without affecting the catalytic activity of H_2_O_2_. Glucose is first oxidized to gluconic acid and H_2_O_2_, and the detection of glucose is achieved through a colorimetric reaction in the presence of TMB. Self-propelled tubular microrobots based on the Marangoni effect are also used for H_2_O_2_ sensing [152]. The surfactant (sodium dodecyl sulfate) is released from the tip of the microrobot, causing a surface tension gradient for propulsion. Meanwhile, HRP is released from the microrobot into the solution to decompose H_2_O_2_, causing TMB to be oxidized into blue color for visual or optical detection. The concentration of H_2_O_2_ is directly proportional to that of the TMB oxidation products, which can also be detected electrochemically by using chronoamperometry. A naked-eye cortisol sensing strategy is developed based on PEDOT/Ni/Pt microtubes modified with anti-cortisol antibody (Fig. 5c) [153]. The microtubes bind fast with HRP tag-labeled cortisol and then make the tetramethylbenzidine and H_2_O_2_ solution display deep blue color, which enables rapid naked-eye sensing (2 min) of cortisol with a low concentration (0.1 μg mL^−1^) and small volume (50 *μ*L). Single-wall carbon nanotube (SW)-Fe_2_O_3_/MnO_2_ microrobots are designed for colorimetric sensing of pollutants (phenylenediamine isomers) in water [154]. The inner MnO_2_ layer catalyzes the decomposition of H_2_O_2_ as fuel to generate oxygen bubbles and hydroxyl radicals, and then the hydroxyl radicals cause the oxidation and dimerization of phenylenediamine isomers to change the color of the solution for colorimetric sensing. The Fe_2_O_3_ particles attached to the outer layer can accelerate fuel decomposition and endow the microrobot with magnetism and reusability.

The reaction between the micro/nanorobot and the target may lead to changes in its own composition or the composition of the environment, which provides information for sensing. Plasmonic-magnetic nanorobots consisting of Fe_3_O_4_/Au/Ag nanoparticles offer a simple and efficient method for the detection of SARS-CoV-2 RNA [54]. The Fe_3_O_4_/Au/Ag nanoparticles self-assemble into rod-shaped microaggregates under rotating magnetic fields, and the formed nanorobot is capable of performing controllable motion. Single-stranded DNA probes are modified on the nanoparticles, which hybridize with the complementary target RNA (Fig. 5d). After the hybridization reaction, the hybridized duplex is released from the nanorobot due to electrostatic repulsion. Quantitative measurement of residual DNA probes on the nanorobots is conducted by using differential pulse voltammetry on screen-printed electrodes, and the decrease in oxidation peak intensity is proportional to the concentration of target SARS-CoV-2 RNA. Pt/Ag_3_VO_4_ Janus particles are reported to have the ability to detect citric acid present in pollutants [155]. The Pt/Ag_3_VO_4_ microrobots perform self-propulsion under the irradiation of UV light without chemical fuels and effectively degrade pollutants (*e.g.*, rhodamine B). The microrobots corrode in the presence of citric acid, in which Ag ions reacted with citric acid to form Ag nanoparticles. Sensing of citric acid is achieved by monitoring the position of the surface plasmon resonance maximum absorption of the released Ag nanoparticles.

### Assisting SERS Sensing

Surface-enhanced Raman scattering is considered a powerful spectroscopic sensing technique capable of detecting low concentrations of analytes with exceptional sensitivity and specificity [167]. Micro/nanorobots actuated in different methods are used to assist in enhancing SERS sensing. For example, in a SERS-based *E. coli* enumeration method, antibody-coated magnetic nanoparticles and gold nanorods bind with *E. coli* through sandwich immunoassay [156]. Magnetic particles allow the separation of the sandwich complexes by magnetic attraction, while gold nanorods act as Raman labels. The nanorods are assembled with 5,5-dithiobis-(2-nitrobenzoic acid) for generating strong Raman signals. A calibration curve is obtained according to the detected SERS signal for calculating the amount of *E. coli* in the sample, and the result has no significant difference from that obtained from the traditional plate count method.

Observation of SERS signals requires a rough conductive material surface, and thus SERS probes are generally passive nanoparticles (such as gold and silver nanoparticles), which can only approach the analytes through passive diffusion [168]. Micro/nanorobot-based SERS probes have been reported in many studies as active analyte enrichment tools to enhance SERS detection. Mei et al. fabricate Au/SiO/Ti/Ag microtubes using rolled-up nanotechnology to enhance SERS signals (Fig. 5e) [157]. The Ag inner layer acts as a catalyst to decompose H_2_O_2_ into water and oxygen for propulsion, while the Au outer layer allows analytes adsorption. Rhodamine 6G as the analyte is a nitrogen-containing cationic dye and adsorbed on the gold surface through electrostatic force and N-Au interaction. Finally, enhanced SERS signals from the microtubes with R6G absorbed are observed. Another H_2_O_2_ fuel-driven bimetallic Au/Ag core/shell nanorod robots demonstrate the capability of SERS detection of explosives [158]. The fast self-propelled motion, as well as the highly reactive and curved Ag surface of the nanorobots, enhances Raman scattering, based on which the real-time SERS detection of trace explosives (picric acid) in solution is realized. A match-like light-powered nanorobot consisting of a core–shell Ag@SiO_2_ nanowire body and a spherical AgCl tail is used for active SERS sensing [159]. Ag@SiO_2_ nanowire serves as the SERS probe, and its inert SiO_2_ shell protects it from contamination and oxidation, which improves the SERS sensing ability via shell-isolated enhanced Raman spectroscopy [169]. Irradiation of UV light on the nanorobot causes the photocatalytic decomposition of the AgCl tail to produce hydrogen ions and chloride ions. The faster diffusion of hydrogen ions than chloride ions results in an overall electric field directed toward the head of the nanorobot, propelling the positively charged nanorobot forward. Under the irradiation of UV light, the nanorobot spontaneously gathered toward the center of the beam, and its motion can be remotely controlled by adjusting the light source. Enhanced Raman signals are obtained in SERS sensing of crystal violet and breast cancer cells with the assistance of the match-like nanorobot. The ultrasound-induced aggregation has been reported to enhance SERs detection [160]. The gold nanorods move randomly in the ultrasound field at a specific frequency to adsorb the analytes and then quickly gather to the position of the acoustic pressure node when reducing the frequency. The ultrasound-driven enrichment method facilitates sensitive and rapid SERS detection of ultra-trace biomolecules (DNA).

The interstitial junctions of plasmonic nanostructures are called “hotspots”, which can significantly enhance the SERS signal of surrounding analytes [170]. In addition to enriching analytes, micro/nanorobots can also be combined with hotspots engineering to improve SERS performance. Qiu and colleagues use nanoimprint and rolling origami techniques to fabricate Au/SiO/Fe hierarchically structured tubular microrobot with high-density plasmonic nanostructure-supported hotspots incorporated on its surface, as shown in Fig. 5f [161]. The microrobots can not only actively enrich analytes through magnetic field-driven motion but also act as “mobile hotspots” to enhance the intensity of SERS signals. Magnetic nanorobots based on silica-coated Fe_3_O_4_ nanoparticles are modified with Ag nanoparticles to serve as SERS probes, where the Ag nanoparticles endow the nanorobots with SERS hotspots [162]. The nanorobots move to the target position under the actuation of external magnetic fields and then rotate to enhance SERS sensing. Moreover, this microrobot has the self-cleaning ability for repeated use and can enter cells through endocytosis to realize SERS sensing of biomolecules in the cytoplasm.

## Conclusion and Outlook

The advantages of employing micro/nanorobots in the field of sensing manifest in several key aspects: First, the active motion of micro/nanorobots increases the mixing effect and contact opportunity with the targets, which enhances mass transport and accelerates the reaction rates for sensing [171, 172]. Besides, their tiny size enables detecting microscopic objects and substances with ultra-small sample volumes [81, 173]. Second, micro/nanorobots can be driven by various mechanisms and exhibit diverse types of motion and deformation capabilities. They can adapt to different environments, and these behaviors can serve as indicators for real-time detection and sensing tasks [174]. Among them, swarming micro/nanorobots with environment adaptability can actively navigate in complex environments and collectively map out local conditions (*e.g.*, pH, temperature, concentration), showing advantageous in remotely sensing tasks [175, 176]. Third, the structure, functionality, and actuation behavior of micro/nanorobots can be designed and customized to meet the specific requirements of different sensing tasks [177, 178]. These tiny robots can be applied as a remote sensor across scales, from DNA, proteins, to cells, and bacteria. The sensing objects vary from inorganic metal ions, chemical pollutants, and organic substances to physical and chemical properties of the environment (*e.g.*, liquid viscosity, ion strength, and flow rate). Fourth, micro/nanorobots enable in situ detection. Direct detection of the original samples can be conducted without complex preparations and operations, which simplifies the detection process and reduces the associated costs [179, 180]. Lastly, in a swarm of micro/nanorobots, each individual is capable of directly or indirectly generating detection signals, thereby reducing the randomness of detection results and enhancing the efficiency and accuracy of detection [96, 181].

We categorize sensing schemes based on the different roles of micro/nanorobots in sensing, including self-generated signal-based sensing, robot behavior-based sensing, targets capture and transport for sensing, and micro/nanorobot-assisted sensing (Tables 1 and 2). Self-generated signal-based sensing relies on detecting the robot-generated signals, such as on–off fluorescence detection of toxins and on–off luminescence for chemical sensing. This type of sensing mechanism is widely applied for sensing tasks in medium, and usually relies on the functionalization of robots since chemistry plays an essential role in determining the physicochemical properties of micro/nanorobots. Behavior-based sensing obtains signals from robot behaviors caused by the presence of targets, in which the behaviors can be directly observed using optical microscopy. Currently, most related research focuses on the motion behavior of robots where the working principle relies on establishing a correlation between the motion speed of robots and the target analytes (*e.g.*, type and concentration). Micro/nanorobot’s deformation-based detection, especially swarm transformation-based sensing is a notable research opportunity since micro/nanorobots interact with surrounding environments through deformation, which is an effective method for acquiring information about the environment [182–185]. Benefiting from active motion and diverse functionalization features, micro/nanorobots can selectively capture and transport target analytes, enabling the separation of target analytes from complex samples and simplifying the sensing process. Micro/nanorobots actuated by different types of methods have been developed to provide promising options for various sensing targets and environments. Micro/nanorobots can also assist in common sensing processes by enhancing the micro-mixing effect, accelerating or triggering required reactions, and providing labels for the target analytes. In Table 3, we compare the advantages and disadvantages of these sensing mechanisms and list the critical factors that should be considered when applying these mechanisms to sensing tasks.

**Table 3:** Advantages and disadvantages of different sensing mechanisms and critical factors that need consideration for sensing tasks

Sensing mechanisms	Advantages and disadvantages	Critical factors and challenges
Self-generated signal	High sensitivity for toxins and chemical sensing	Integrated sensing and actuation capabilities for targeted sensing
	Relatively easy to process signal	Fast signal processing scheme
	Require robot’s functionalization and modification	Require biocompatible and biodegradable materials
Robot behavior	Directly observe robot behaviors in different environments	Require a real-time robot behavior recognition scheme
	No complex functionalization involved	Require a correlation model between robot behavior and target analytes
	Relatively low sensitivity and larger applications range	Advanced robot design to enhance cross sensitivity
Targets capture and transport	On-demand manipulation of targeted analytes	Require a closed-loop operation system
	Separation of target analytes from samples	High manipulation precision
	Relatively low efficiency for dealing a large amount of sample	Require batch operation or collective control strategy
Micro/nanorobot-assisted sensing	Provide enhanced mixing effect to increase sensing efficiency	Require locally controlled active behavior
	Accelerate reactions by active control robots	Trigger required actions in a controlled manner
	The materials of robot may interference sensing results	Comparison study with and without the robot is required

**Fig. 6:**
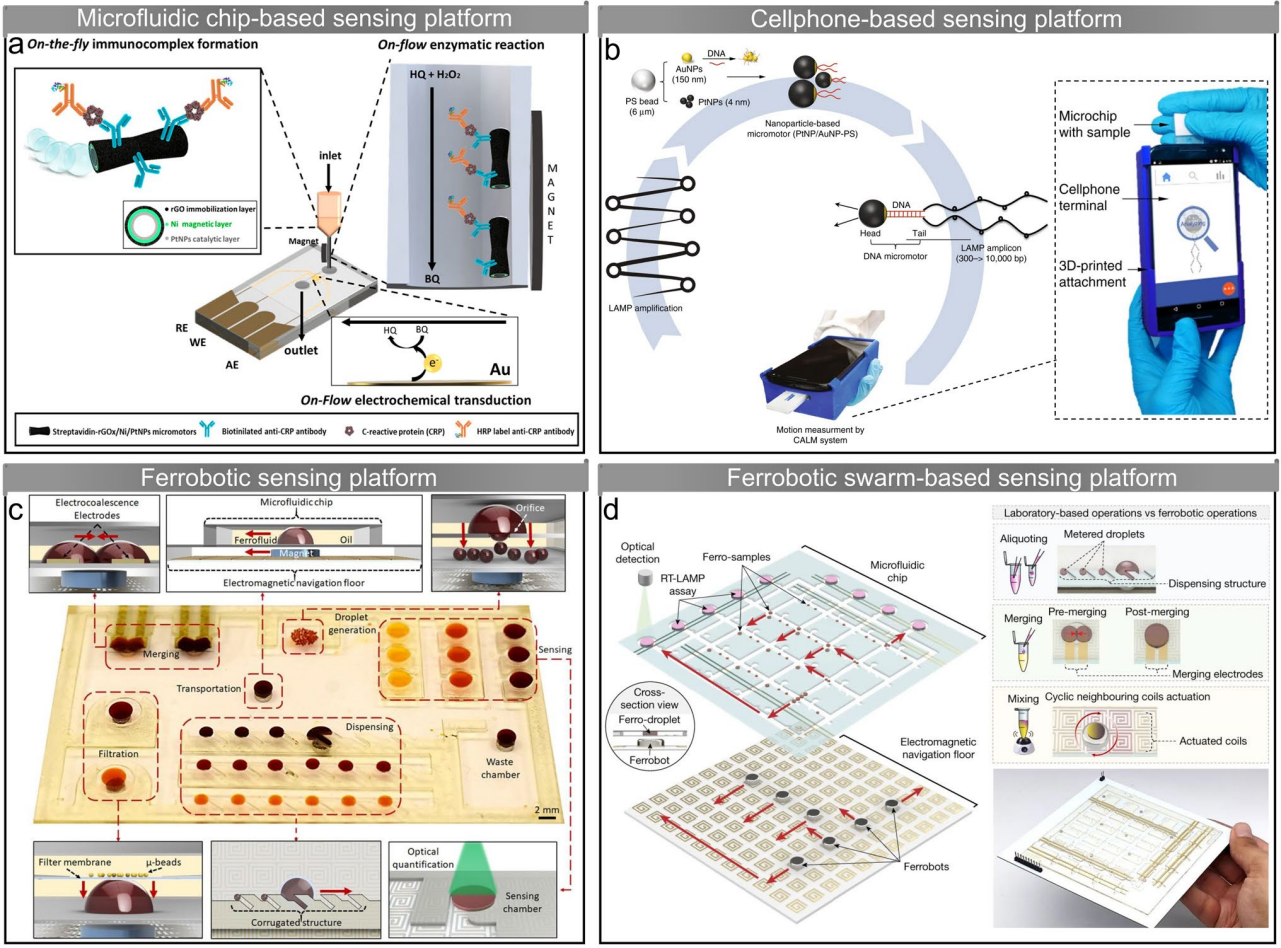
Micro/nanorobot-based sensing platforms. **a** Magnetic rGO-based microrobots are integrated into electrochemical microfluidic chips for CRP sensing [145]. Copyright (2020) American Chemical Society. **b** DNA-engineered Pt microrobots integrated into a cellphone system for HIV-1 sensing [90]. Copyright (2018) Springer Nature. **c** Multifunctional ferrobotic system for fully automated assay [186]. Copyright (2020) The American Association for the Advancement of Science. **d** Automated viral sensing platform based on ferrobotic swarms [10]. Copyright (2022) Springer Nature

Although the fundamental sensing principles remain unchanged, the introduction of micro/nanorobots improves the sensing efficiency and sensitivity with the potential for application in various traditional detection techniques. Although significant progress has been made in the research on micro/nanorobot-based sensing, most of the research work focuses on the design of micro/nanorobots and the exploration of sensing mechanisms, which only stays at the proof-of-concept stage. Building micro/nanorobot-based sensing platforms for wide practical applications is an essential step in the development of this field. Some studies have reported the integration of micro/nanorobots into microchip systems. For example, polymer/Ni/Pt microtubes are used to sequentially capture and transport target antigens and secondary antibodies in a microchip [187]. The system greatly simplifies and accelerates the detection process, enabling the sensing of various analytes, including proteins and bacteria, to be carried out efficiently and conveniently. In an on-chip concentrating system, self-propelled catalytic microrobots functionalized with streptavidin selectively bind with biotinylated components [188]. The microrobots are trapped by V-shaped and ratchet-shaped physical boundaries when moving in a heart-shaped microfluidic chip, and the concentration process is then completed. As shown in Fig. 6a, rGO-based magnetic microrobots are coupled to an electrochemical microfluidic chip for CRP sensing [145]. The antibody-functionalized microrobots perform sandwich immunocomplex with CRP and HRP-labeled anti-CRP secondary antibody in an external reservoir. Subsequently, they are magnetically retained in the metallic channel, where enzymatic substrates flow through, facilitating the process of electrochemical detection. The microrobotic system combined with microfluidic electrochemical detection technology allows automated, accurate, and rapid CRP detection with no loss of performance and sensitivity compared to other work [134] while achieving miniaturization and integration for on-site/bedside clinical analysis. In addition to microchips, micro/nanorobots are integrated into cellphone systems to build fast, portable, and low-cost disease diagnosis tools. Two sensing micro/nanorobots we introduced in Sect. 3, *i.e.*, Pt-base microrobots for Zika virus [80] and HIV-1 RNA [90] sensing, are combined with cellphone-based optical sensing technology. Figure 6b shows the schematic diagram and actual image of the loop-mediated isothermal amplification and microrobot motion (CALM) cellphone system for HIV-1 sensing [90]. Qualitative HIV-1 sensing with a threshold of 1000 virus particles mL^−1^ and high specificity (99.1%) and sensitivity (94.6%) is achieved using this system without the need for expensive fluorescent optical components. Emaminejad et al. construct an individually addressable ferrobotic system and apply it within a microfluidic architecture framework, as shown in Fig. 6c [186]. Ferrofluid droplets, serving as cargo carriers, are manipulated by the electromagnetic navigation floor and millimeter-scale permanent magnets to perform a variety of microfluidic operations, such as droplet dispensing, generation, merging, and filtering. This multifunctional ferrobotic system is highly robust and enables fully automated quantification of analytes, such as active matrix metallopeptidases in human plasma, through cross-cooperation of ferrobots. Recently, the same group has developed an automated viral sensing platform based on ferrobotic swarms, in which magnetic nanoparticle-spiked droplets are precisely and robustly manipulated by a swarm of individually addressable millimeter-sized magnets (Fig. 6d) [10]. Fluidware, hardware, and software are integrated into the platform, enabling the detection of samples under the guidance of a square matrix pooled testing algorithm. The whole system is only palm-sized and capable of batch sample processing, including transfer, aliquoting, merging, mixing, and heating of sample droplets. This platform is expected to be used as a commercial virus detection tool, which can reduce the cost of reagents and instruments by 10–300 times and three orders of magnitude, respectively. These sensing platforms integrated with micro/nanorobots represent the future direction of development for micro/nanorobot-based sensing, aiming for practicality and commercialization. Therefore, there is a need to develop advanced sensing platforms that fully leverage the advantages of micro/nanorobots, with the potential to revolutionize traditional sensing methods.

**Fig. 7:**
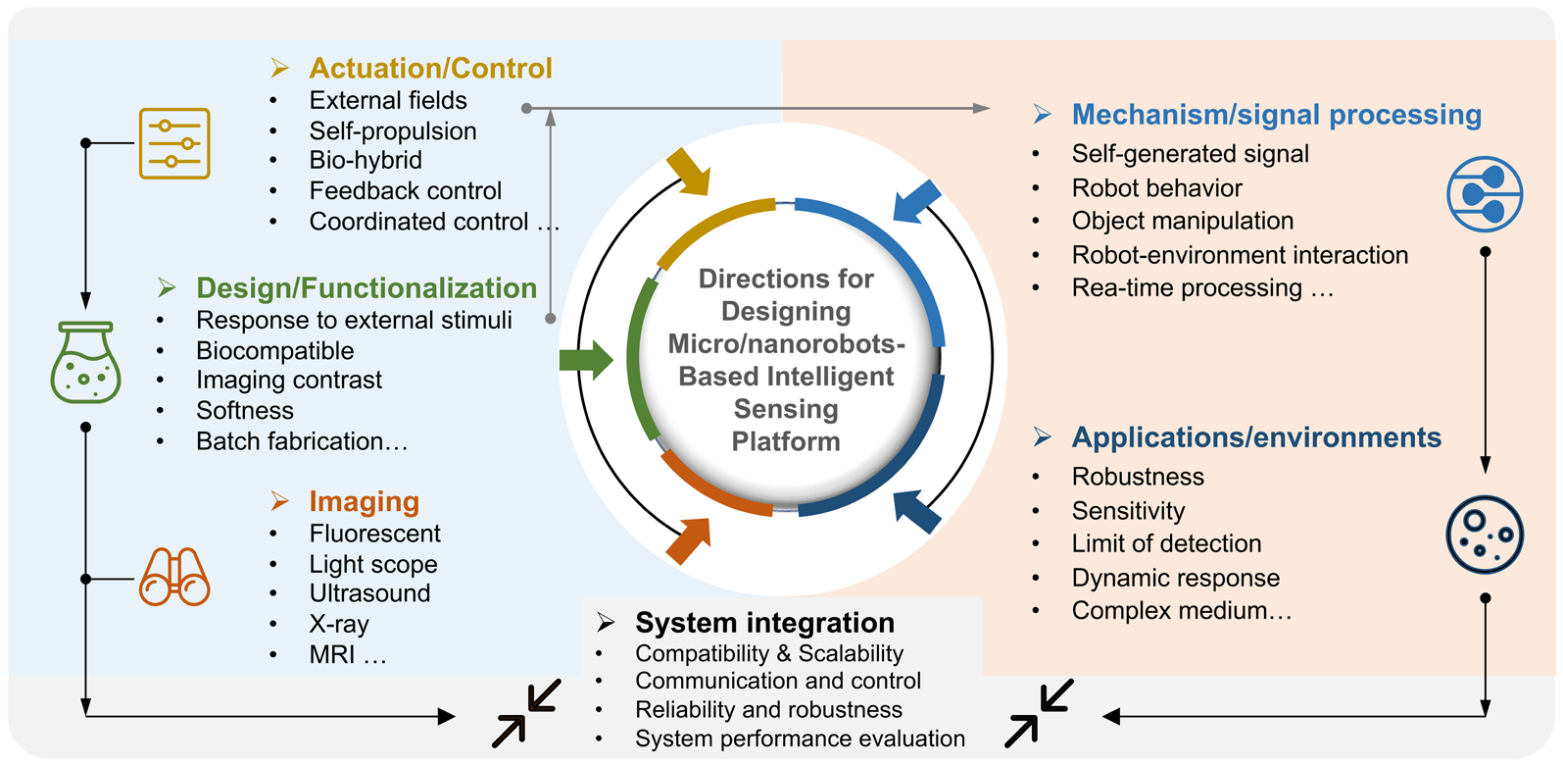
Key factors for designing micro/nanorobots-based intelligent sensing platforms for practical applications

Most micro/nanorobots-based sensing and detection are currently conducted in vitro. However, there are challenges in controlling and imaging micro/nanorobots within a living body, where the precision of signal detection and observation of robot behavior is hampered by the complex operational environment. Additionally, strategies for the controllable deployment and recycling of the robots need further investigation. Leveraging micro/nanorobots for in vivo monitoring, especially within the complex environments of a living organism, enables real-time acquisition of in vivo information through robot-generated signals and behaviors. This capitalizes on the advantages of their small size and controllable motion. It allows us to gather information about target locations without involving complex procedures, benefiting early disease diagnosis. However, challenges exist in implementing chemical fuel-driven micro/nanorobots for in vivo applications due to potential biocompatibility issues. These challenges can be addressed by developing alternative propulsion methods, such as ultrasound or magnetic propulsion, and employing biocompatible materials (*e.g.*, natural biomimetic templates) for robot fabrication. Additionally, achieving high imaging contrasts is essential for real-time in vivo observation of micro/nanorobots, which requires the integration of diverse medical imaging modalities to visualize the mobile micro/nanorobots and obtain signals for sensing [189–195]. After completing their tasks, strategies for recycling, metabolizing, or degrading the robots should be included in the system design [196]. Currently, most micro/nanorobots are designed for sensing a single target. Developing multifunctional sensing platforms will enable convenient and rapid detection of multiple targets. To overcome these challenges, future efforts will focus on building intelligent sensing platforms that require a series of technological breakthroughs in micro/nanorobots and collaboration across diverse disciplines (Fig. 7). The design and functionalization of micro/nanorobots should be considered as a priority since they play an essential role in defining the suitable actuation type and imaging modality. Given the sensing task and manipulation environment, a strategy for the sensing mechanism and signal processing should be evaluated, which also guides the robot system design. From the perspective of system integration, a trade-off between different factors should be considered to obtain an efficient and sensitive system with a certain level of automation. By continually evaluating the system’s performance for each sensing case, the sensing platform will keep learning and improving itself toward a more intelligent level. With continuous efforts to improve robotic control, functionalization, sensing mechanisms, and system integration, we believe that the research achievements in the lab will lead to widespread real-world sensing applications of micro/nanorobots, significantly enhancing our quality of life.
